# A facile aqueous production of bisphosphonated-polyelectrolyte functionalized magnetite nanoparticles for pH-specific targeting of acidic-bone cells†

**DOI:** 10.1039/d1ra09445a

**Published:** 2022-03-11

**Authors:** Md. Abdur Rahman, Bungo Ochiai

**Affiliations:** Department of Chemistry and Chemical Engineering, Graduate School of Science and Engineering, Yamagata University 4-3-16, Jonan Yonezawa Yamagata 992-8510 Japan ochiai@yz.yamagata-u.ac.jp; Polymer Colloids and Nanomaterials Lab, Department of Chemistry, Faculty of Science, Rajshahi University Rajshahi 6205 Bangladesh

## Abstract

Bone malignancy treatment is being hindered due to the insufficient selectivity of therapeutic nanoparticles towards malignant bone sites. Polyelectrolyte functionalized magnetic nanoparticles having dually specific pH-sensing ability and bisphosphonate moieties, can be an effective solution for selective targeting of bone malignancies. First, polyelectrolyte was prepared *via N*-carboxycitraconyzation of chitosan (NCCS) followed by successive functionalization with alendronic acid (AL) and fluorescein isothiocyanate (FITC). Then, Fe_3_O_4_-NCCS-FITC-AL nanoparticles were synthesized by a facile one-step microwave-assisted aqueous method *via in situ* surface functionalization. The formation, crystal structure, and surface conjugation of Fe_3_O_4_ nanoparticles with polyelectrolytic stabilizer were confirmed by Fourier transform infrared spectroscopy, X-ray diffraction, and thermogravimetric analyses. Synthesized Fe_3_O_4_-NCCS-FITC-AL nanoparticles were superparamagnetic, colloidally stable and highly hemocompatible under physiological conditions. Moreover, at pH 5.0, Fe_3_O_4_-NCCS-FITC-AL nanoparticles formed a precipitate due to inversion of their surface charge. This pH-dependent charge-inversion drastically changed the interactions with erythrocytes and bones. Selective membranolysis of erythrocytes occurred at pH 5.0. The designed nanoparticles showed enough potential for selective targeting of pathological bone sites in early-stage magnetofluorescent imaging and as a therapeutics carrier to treat malignant bone diseases.

## Abstract 2

Synthesis of a bisphosphonated polyelectrolytic stabilizer to *in situ* fabricate and functionalize Fe_3_O_4_ nanoparticles and their pH-dependent hemolysis and bone-cell adhesion.

## Graphical abstract

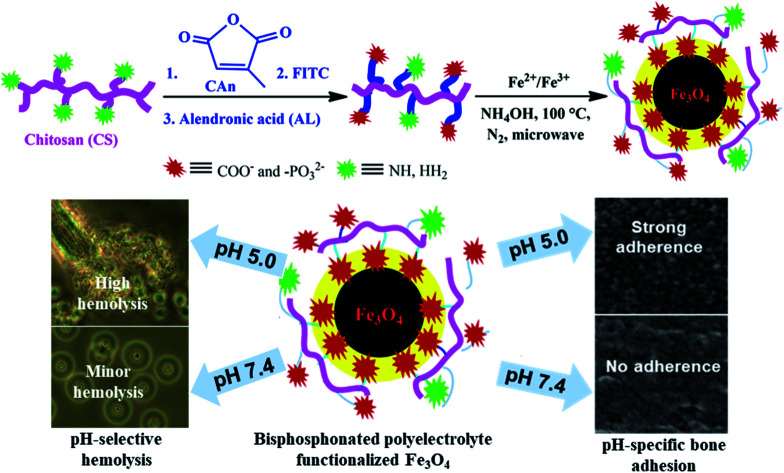

## Introduction

Metastatic and metabolic bone malignancies occur in a wide range of ages including children, and the symptoms such as severe bone pain are distressing to various patients.^1^ Current therapeutics like those based on selective estrogen receptor modulators and bisphosphonates are poorly bioavailable, require frequent and high dosing, and cannot precisely target disease sites.^2^ Therefore, these drugs are often ineffective due to their high systemic toxicity and off-targeted side effects including arterial pain, osteonecrosis of the jaw, musculoskeletal pain, and ulcers.^3,4^ Various nanomaterials, such as polymeric micelles,^5,6^ liposomes,^7^ and gold,^8^ and magnetic nanoparticles with targetable functional moieties are being used to target the pathological sites for diagnosis and therapy of bone malignancies.^6–15^ Among them, magnetic iron oxide nanoparticles (MIONPs) have attracted significant research attention. For instance, MIONPs have been used for detoxification of biofluids,^10,11^ drug targeting vehicles,^13–15^ thermal ablation of tumor cells *via* apoptosis,^16,17^ and contrast agents for magnetic resonance imaging.^13–15^ MIONPs can be synthesized through nonaqueous routes like solvothermal, microemulsion, high-temperature decomposition of organo-iron precursors, and aqueous paths, such as hydrothermal and coprecipitation methods. However, non-functionalized MIONPs are highly prone to aggregate because of their strong magnetic attraction forces and high surface energy. The colloidal instability leads to opsonization and fast recognition by macrophages and mononuclear phagocytes. These unmodified MIONPs are rapidly removed through the reticuloendothelial system (RES).^13,15,18^ So, the RES escaping is a vital challenge to any MIONPs used in diagnosis and therapy. In addition, the size, shape, surface functionality, nature of surface charge and colloidal stability of MIONPs also affect the evasion of RES. The RES engulfment can be minimized by prolonging circulation time of MIONPs *via* functionalization with mimicking ligands that masks phagocyte surfaces as well as coating their surface with hydrophilic polymers.^14^ Therefore, appropriate surface functionalization of MIONPs is essential for bio-related applications because the functional moieties first encounter with biological entities.^19^ So far, various materials having different functional moieties like poly(ethylene glycol) (PEG), poloxamer, polyvinyl alcohol, polyaminoacid, carbohydrates, carboxylate and bisphosphonated polymers have been introduced on the surface of MIONPs.^20–29^ As a result, surface functionalized particles having relatively wider size of 100–200 nm can easily be accumulated and retained in the targeted sites due to their enhanced permeability and retention effect.

Here, bisphosphonate moiety consisting of P–C–P bond is investigated as a bone targeting, pH-sensing, and dispersing milieu for MIONPs. Because, small organic molecules of bisphosphonates have been found useful to treat various bone malignancies like chondrosarcoma, osteosarcoma, Ewing's sarcoma, inflammation of bones due to rheumatoid arthritis, periodontal disease, and so on.^3,15^ They are also efficient to inhibit bone resorption due to their ability to bind bone mineral by bi- and tridentate ligation to Ca^2+^ ions *via* osteoclastic differentiation and apoptosis.^3,15^ Until now, a number of targeting nano-systems based on bisphosphonates have been developed, and some of them have been tested preclinically for bone metastasis.^3,9,10,12–15,30,31^

But bisphosphonates employed for bone-targeting are non-specific, and as a result, they target both healthy and malignant bone tissues without selectivity resulting in poor performances of therapeutics and adverse side effects.^3^ Therefore, development of synthetic methods for MIONPs with targeting ability towards malignant bone cells *via* facile and biosafe procedures is still a great challenge. pH-responsive systems may introduce selectivity to therapeutics based on bisphosphonate-functionalized MIONPs for metastatic bone diseases as well as primary bone malignancies.^32–36^ Early-stage detection of bone malignancy enables more options for better treatments, resulting in higher survival rates and enhance quality of life.^37–47^ Ferreira *et al.* developed bone-targetable pH-sensitive bisphosphonated liposomes and evaluated their cytotoxicity, cardiotoxicity, biodistribution, and used them for treating bone metastasis.^7^ Chen *et al.* reported a bisphosphonated amphiphilic hyperbranched polymer and PEG-based micelles for targeting drugs to maligned bone cells.^6^ Stewart *et al.* reported bisphosphonated acid-sensitive drug-loaded micelles for the treatment of osteosarcoma.^5^ Au *et al.* designed pH-responsive folate conjugated nanoscale MOF bearing bisphosphonate moieties for treating a tumor.^34^ The pH responsibility of 1,2-dioleoyl-*glycero*-3-phosphatiethanolamine and cholesteryl hemisuccinate, ester/amide, hydrazone and phosphonate moieties are the key of these pH-sensitive systems.

To the best of our knowledge, there is no report on acid-sensible charge-inversional bisphosphonated MIONPs for targeting and treatment of malignant bone cells. The present research focuses on a facial aqueous synthetic route to synthesize a novel magnetic nano-biosystem using a polyelectrolytic stabilizer for selective targeting of acidic bone cells. The polyelectrolyte stabilizer was first prepared *via N*-carboxycitraconyzation of chitosan (NCCS) followed by successive functionalization. Then, *in situ* synthesis of Fe_3_O_4_ and concomitant functionalization was carried out to obtain Fe_3_O_4_-NCCS-FITC-AL nanoparticles. The designed nanoparticles would enhance therapeutic efficacy and minimize systemic and off-targeted side effects. In addition, the conjugated polyelectrolytic-NCCS-FITC-AL layer is supposed to improve hemocompatibility, biocompatibility, acid-sensing charge-stealth ability, and selective targeting ability of the MIONPs towards malignant bone sites. We selected AL molecule as a bisphosphonate for bone-targeting component, carboxylate moieties for the acid-sensing, and CS for the biocompatible and reactive scaffold of the polyelectrolytic coating. Fluorescein isothiocyanate (FITC) was employed as a fluorescent probe for facile imaging and detection of materials. The formation, crystal structural, colloidal stability, pH-sensitive charge-inversion ability, hemocompatibility, magnetic property of the nanoparticles were confirmed and evaluated. Erythrocytes membranolytic efficiency, fluorescent properties as well as selectivity of the Fe_3_O_4_-NCCS-FITC-AL nanoparticles towards bone mineral (HAp) and a native bone sample under physiological and acidic conditions were also studied.

## Results and discussion

### Synthesis and characterization of polyelectrolytic NCCS-FITC-AL stabilizer

**Scheme 1:**
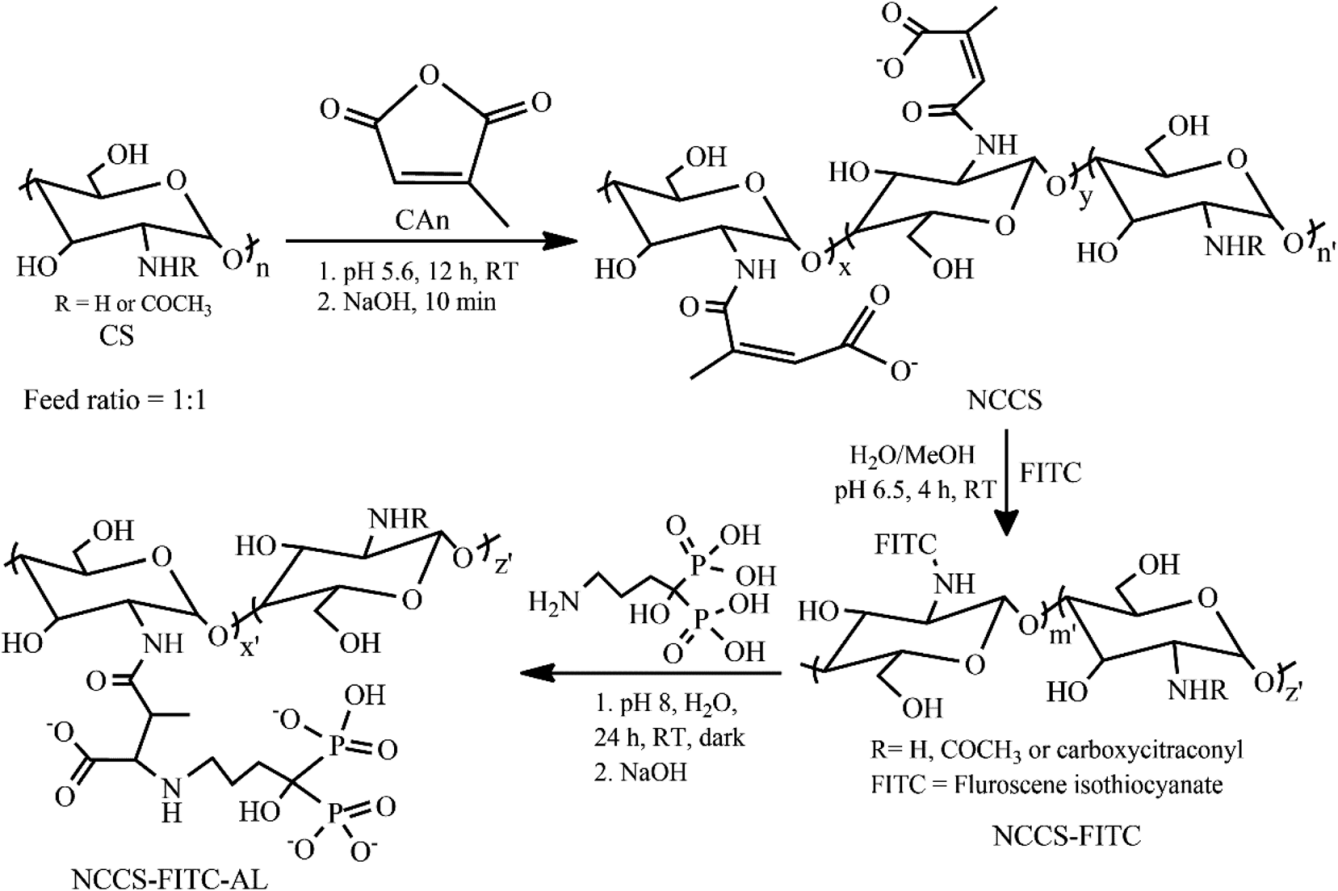
Synthesis of NCCS-FITC-AL polyelectrolytic stabilizer from CS.

A simple three-step procedure was used to synthesize polyelectrolytic NCCS-FITC-AL (Scheme 1). In the first step, NCCS was synthesized by partial amidation of the primary amine groups in CS with citraconic anhydride (CAn) for improving dispersity in aqueous media with pH ranging from acidic to weakly basic by the carboxy group. The carboxy group in NCCS is easily transformable to the mono-Na salt *via* neutralization with aqueous NaOH. Synthesized NCCS is an amorphous fine white powder (Fig. S1a†), whose dispersity was dramatically improved than CS. It was fully dispersible in neutral deionized distilled water (DDW) due to the addition of hydrophilic carboxylate moieties to CS. The fluorescent group was conjugated in the second step through the conventional nucleophilic addition of the primary amine moieties of NCCS to the isothiocyanate groups of FITC. The resulting product is a yellow-colored fine powder (Fig. S1b†). The NCCS-FITC chain possesses the primary amine, hydroxy, carboxy, and unsaturated ester groups that can be easily conjugated with suitable reagents. Among them, the highly electron-deficient unsaturated ester groups are very prone to nucleophilic addition of amines even under slightly acidic aqueous environments. In the last step, the bisphosphonate moieties were partially introduced *via* aqueous aza-Michael addition of the primary amine moieties of AL to the alkenyl groups of NCCS-FITC under mildly acidic conditions to form the desired polyelectrolyte, NCCS-FITC-AL. The formed polyelectrolyte was also a light-yellow-colored (Fig. S1c†) very fine powder that was also completely dispersible in DDW.

The successful synthesis of NCCS-FITC-AL was confirmed by energy-dispersive X-ray (EDX) (Fig. S2†), Fourier transform infrared (FTIR) (Fig. S3†), ^1^H NMR (Fig. S4†), and UV-vis (Fig. S5†) spectroscopic studies. The EDX spectroscopic analysis showed the respective elements present in the NCCS, NCCS-FITC, and NCCS-FITC-AL structures. Especially Na and P originated from the carboxylate and bisphosphonate moieties. The characteristic bands appeared in the FTIR spectra of NCCS, NCCS-FITC, and NCCS-FITC-AL indicated the introduction of the carboxy (*ν*_C=O_ at 1406 cm^−1^), fluorescent (*ν*_C=C_ at 1450–1600 cm^−1^), and bisphosphonate (*ν*_P=O_ at 1108 cm^−1^) moieties on CS (Fig. S3†). The ^1^H NMR spectroscopic analysis of CS, NCCS, NCCS-FITC, and polyelectrolytic NCCS-FITC-AL further confirmed the modifications of CS by the signals indicating the introduction of the alkenyl and methylene groups (Fig. S4†). The introduction ratio of the *N*-citraconyl group was calculated to be 44% to the amine groups from the integral ratio of the peaks assigned to the acetyl and alkenyl protons of NCCS. The introduction ratio of the bisphosphonate moieties was calculated to be 53% with respect to the *N*-carboxycitraconyl group. The presence of the FITC residues in the NCCS-FITC-AL structure was also confirmed by an absorption band at 495 nm observed in the UV-vis spectrum (Fig. S5†).

### *In situ* aqueous synthesis of Fe_3_O_4_ nanoparticles and their concomitant surface functionalization with polyelectrolytic NCCS-FITC-AL

**Scheme 2:**
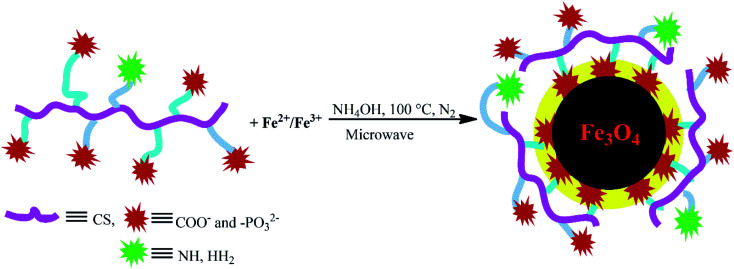
Schematic illustration for *in situ* aqueous synthesis of Fe_3_O_4_ nanoparticles and their concomitant surface functionalization with polyelectrolytic NCCS-FITC-AL by MW-assisted one-step coprecipitation-coating method.

The charge-inversional Fe_3_O_4_-NCCS-FITC-AL nanoparticles were prepared by a modified coprecipitation method (Scheme 2). This method involved with *in situ* production of Fe_3_O_4_ nanoparticles and concomitant surface functionalization with polyelectrolytic stabilizer, NCCS-FITC-AL, *via* nucleation, seed generation, and particle growth.^49,50^ When the solutions of the Fe(ii) and Fe(iii) precursors were added to the dispersion of NCCS-FITC-AL under N_2_, the iron ions were accumulated to the carboxy and bisphosphonate moieties. Rapid nucleation of Fe_3_O_4_ particles was occurred by the addition of aqueous ammonia solution through hydroxylation and dehydration of the localized iron ions. Then, the dispersion was transferred to a microwave (MW) oven, where MW was irradiated to grow particles at 100 °C for 30 min. In this method, NCCS-FITC-AL served as a scaffold to control the growth of the nanoparticles. NCCS-FITC-AL can also limit the active mass transfer of iron compounds to the surface of the Fe_3_O_4_ nanoparticles from the reaction medium through the polydentate coordination of the carboxylate and bisphosphonate moieties to the Fe atoms on the Fe_3_O_4_ surface. In addition, NCCS-FITC-AL stabilized the *in situ* prepared Fe_3_O_4_ with the electrostatic repulsion of the ionic moieties on the outer surface not involved to the ligation on the Fe_3_O_4_ surface.

### Characterization of Fe_3_O_4_-NCCS-FITC-AL nanoparticles

**Fig. 1:**
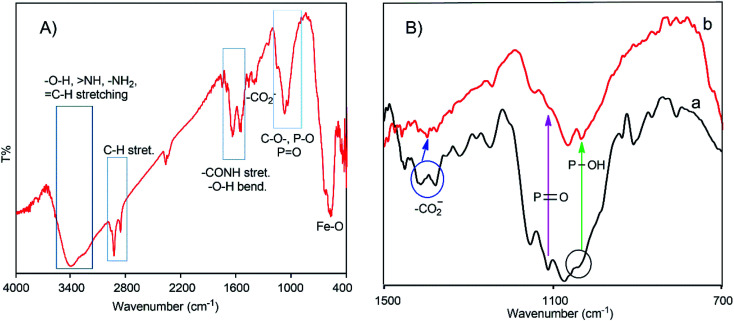
Whole (A) and magnified (700–1500 cm^−1^ region) (B) FTIR spectra of Fe_3_O_4_-NCCS-FITC-AL nanoparticles (a) and NCCS-FITC-AL (b).

The formation of the charge-inversional Fe_3_O_4_-NCCS-FITC-AL nanoparticles was confirmed by FTIR and EDX (Fig. S6a†) spectroscopic, and thermogravimetric analyses (Fig. S6c†). A well-washed and fully dried powder sample was used for the FTIR spectroscopic analysis (Fig. 1A). The FTIR spectrum of Fe_3_O_4_-NCCS-FITC-AL shows two bands at 548 and 573 cm^−1^ assignable to the stretching vibrations of the Fe–O bond in typical Fe_3_O_4._ It did not show the Fe–O bands at the higher wavenumber region originating from Fe_2_O_3_ observable in 600–690 cm^−1^ (Fig. S6d†).^13^ This result demonstrates that Fe_3_O_4_ nanoparticles were predominantly formed over Fe_2_O_3._ The oxidation of the iron(ii) ions in Fe_3_O_4_ did not occur by the phosphonate groups.^13,51^ The characteristic bands of the phosphonate group generally appeared in the range of 900–1200 cm^−1^. Therefore, the precise locations of the bands of P=O and P–O bonds are quite difficult to assign as they are overlapping with other bands such as those of the C–O and C–O–C moieties in NCCS-FITC-AL. Hence, the conjugation of functional groups like carboxylate and phosphonate to the Fe_3_O_4_ surface was confirmed by careful analysis on the comparison of the magnified spectra of Fe_3_O_4_-NCCS-FITC-AL and NCCS-FITC-AL (Fig. 1B, 700–1500 cm^−1^). The stretching vibration for P=O in the phosphonate moieties in NCCS-FITC-AL was observed at 1108 cm^−1^. When the phosphonate groups interacted with iron atoms, the stretching vibration for P=O significantly changed from 1108 to 1133 cm^−1^. The stretching bands of P–OH were also significantly shifted from 1040 to 1033 cm^−1^ and 906 to 902 cm^−1^. In addition, a new peak appeared at 1030 cm^−1^ assignable the stretching vibration mode of Fe–O–P indicating an effective conjugation of the phosphonate moieties with the surface iron atoms on the Fe_3_O_4_ nanoparticles.^13,15,32^ On the other hand, the stretching vibration bands of the carbonyl group in the carboxylate ions were also shifted from 1410 to 1396 cm^−1^ and 1376 to 1372 cm^−1^ due to the bidentate chelation to iron atoms on the surface of the Fe_3_O_4_ nanoparticles. In addition, the peak intensity of the absorption of the carboxylate ion was significantly lowered than that of the phosphonate structure, which demonstrated dominant coordination of the carboxylates to the Fe_3_O_4_ nanoparticles, and the bisphosphonate moieties mostly remained intact for targeting. The other peaks related to the structure of NCCS-FITC-AL also appeared in shifted positions. For instance, some medium–strong bands were observed at 1645, 1625, and 1617 cm^−1^, and these bands are assignable to the stretching bands of amide-I and II and C=C overlapped with the bending bands of the –OH, <svg xmlns="http://www.w3.org/2000/svg" version="1.0" width="10.400000pt" height="16.000000pt" viewBox="0 0 10.400000 16.000000" preserveAspectRatio="xMidYMid meet"><metadata> Created by potrace 1.16, written by Peter Selinger 2001-2019 </metadata><g transform="translate(1.000000,15.000000) scale(0.011667,-0.011667)" fill="currentColor" stroke="none"><path d="M80 1160 l0 -40 40 0 40 0 0 -40 0 -40 40 0 40 0 0 -40 0 -40 40 0 40 0 0 -40 0 -40 40 0 40 0 0 -40 0 -40 40 0 40 0 0 -40 0 -40 40 0 40 0 0 -40 0 -40 40 0 40 0 0 80 0 80 -40 0 -40 0 0 40 0 40 -40 0 -40 0 0 40 0 40 -40 0 -40 0 0 40 0 40 -40 0 -40 0 0 40 0 40 -40 0 -40 0 0 40 0 40 -80 0 -80 0 0 -40z M560 520 l0 -40 -40 0 -40 0 0 -40 0 -40 -40 0 -40 0 0 -40 0 -40 -40 0 -40 0 0 -40 0 -40 -40 0 -40 0 0 -40 0 -40 -40 0 -40 0 0 -40 0 -40 -40 0 -40 0 0 -40 0 -40 80 0 80 0 0 40 0 40 40 0 40 0 0 40 0 40 40 0 40 0 0 40 0 40 40 0 40 0 0 40 0 40 40 0 40 0 0 40 0 40 40 0 40 0 0 80 0 80 -40 0 -40 0 0 -40z"/></g></svg> NH, and –NH_2_ moieties. Two distinct sharp bands were visible at 2922 and 2853 cm^−1^ assignable to the C–H stretching of the CH_2_ groups in the amino sugar units and AL. A broad band appeared at 3000–3500 cm^−1^ is assignable to the stretching vibrations of O–H, NH, –NH_2,_ and =C–H bonds.

### Morphology and core-structure of Fe_3_O_4_-NCCS-FITC-AL nanoparticles

**Fig. 2:**
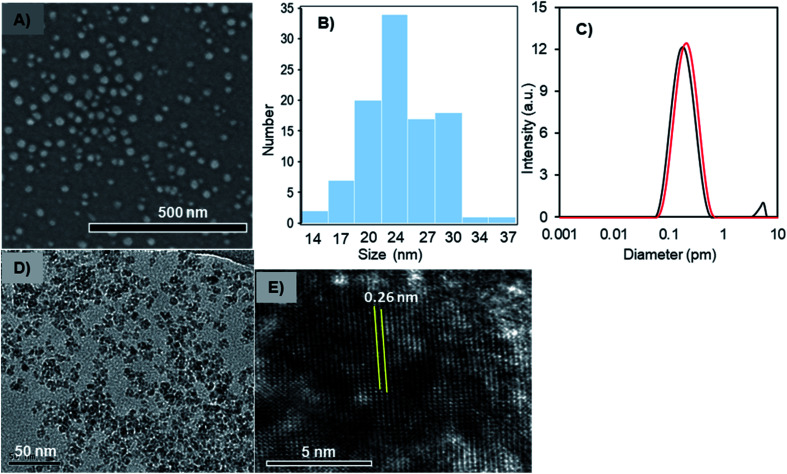
(A) SEM image, (B) histogram of diameters of particles in the SEM image, (C) DLS profiles (red, after MW-treatment; black, before MW-treatment), (D) TEM image, (E) HRTEM image of the Fe_3_O_4_-NCCS-FITC-AL nanoparticles.

The solid- and liquid-state morphologies and core-structure of the Fe_3_O_4_-NCCS-FITC-AL nanoparticles were analyzed by scanning electron microscopy (SEM), transmission electron microscopy (TEM), dynamic light scattering (DLS), and X-ray diffraction (XRD) (Fig. 2). The SEM image (Fig. 2A) revealed that Fe_3_O_4_-NCCS-FITC-AL nanoparticles are singly dispersed and almost spherical. The average diameter of Fe_3_O_4_-NCCS-FITC-AL nanoparticles was calculated to be 27 nm, and the size-distribution histogram is shown in Fig. 2B. The well-dispersed state demonstrates that polyelectrolytic NCCS-FITC-AL effectively covering the magnetic-Fe_3_O_4_ core reduced the inter-particle attractions through steric and electrostatic repulsions. The morphology of the core of the Fe_3_O_4_-NCCS-FITC-AL nanoparticles was analyzed by TEM (Fig. 2D). The TEM image revealed almost similar shapes of the nanoparticles, while the obtained average core size is one-fourth of the size revealed by the SEM image showing the morphology of the particles covered by the polymeric coating in a dried state. Fig. 2C shows the DLS profiles of the Fe_3_O_4_-NCCS-FITC-AL nanoparticles before and after microwave treatment. The average hydrodynamic diameter (*D*_h_) of the nanoparticles was estimated to be 160 nm (black) with a PDI of 0.30. The *D*_h_ of the Fe_3_O_4_-NCCS-FITC-AL nanoparticles was increased from 160 to 190 nm (red) after MW treatment and the PDI was also significantly lowered from 0.30 to 0.18. The increment of the size is ascribable to the growth of the particles during the MW-irradiation. The difference between the sizes observed by DLS and SEM is ascribable to the thickness of the swollen layer of NCCS-FITC-AL^52^ and the Brownian motion of the particles. XRD analysis was conducted to determine the crystal structure of the core component inside the Fe_3_O_4_-NCCS-FITC-AL nanoparticles. The XRD profiles of dried, uncoated Fe_3_O_4_ and Fe_3_O_4_-NCCS-FITC-AL nanoparticles are illustrated in the 2*θ* range of 10–70° (Fig. S6b†). The XRD profile of uncoated Fe_3_O_4_ showed six reflections centered at 30.1°, 35.5°, 43.3°, 53.6°, 57.4°, and 62.8° assignable to the (220), (311), (400), (422), (511), and (440) lattice planes, respectively, of the cubic spinel Fe_3_O_4_ with the lattice constant *a* = 8.4040 Å (JCPDS 85-1436).^53^ Almost identical patterns were observed for the Fe_3_O_4_-NCCS-FITC-AL nanoparticles without any additional reflections assignable to any other iron species generated *via* oxidation during the preparation and storage. The lattice constant for Fe_3_O_4_-NCCS-FITC-AL nanoparticles was calculated to be 8.3895 Å, which is closer to the lattice constant of bulk magnetite (8.3960 Å), indicating that the *in situ* prepared Fe_3_O_4_ nanoparticles were effectively stabilized by polyelectrolytic NCCS-FITC-AL without altering the crystal structure of the Fe_3_O_4_ core.^13,53^ This result manifests the presence of single-phase-core-Fe_3_O_4_ inside the Fe_3_O_4_-NCCS-FITC-AL nanoparticles. Crystallinity and structure of the core component of the Fe_3_O_4_-NCCS-FITC-AL nanoparticles were further analyzed by high-resolution TEM (HRTEM). Fig. 2E shows a typical HRTEM image showing vividly clear lattice fringes. The interplanar distance is 0.26 nm, which is assignable to the (311) lattice spacing of cubic spinel Fe_3_O_4_. FTIR, XRD data and TEM analysis indicated the presence of spinel Fe_3_O_4_ rather than the defective spinel γ-Fe_2_O_3_ inside the Fe_3_O_4_-NCCS-FITC-AL nanoparticles.

### Magnetism of Fe_3_O_4_-NCCS-FITC-AL nanoparticles

**Fig. 3:**
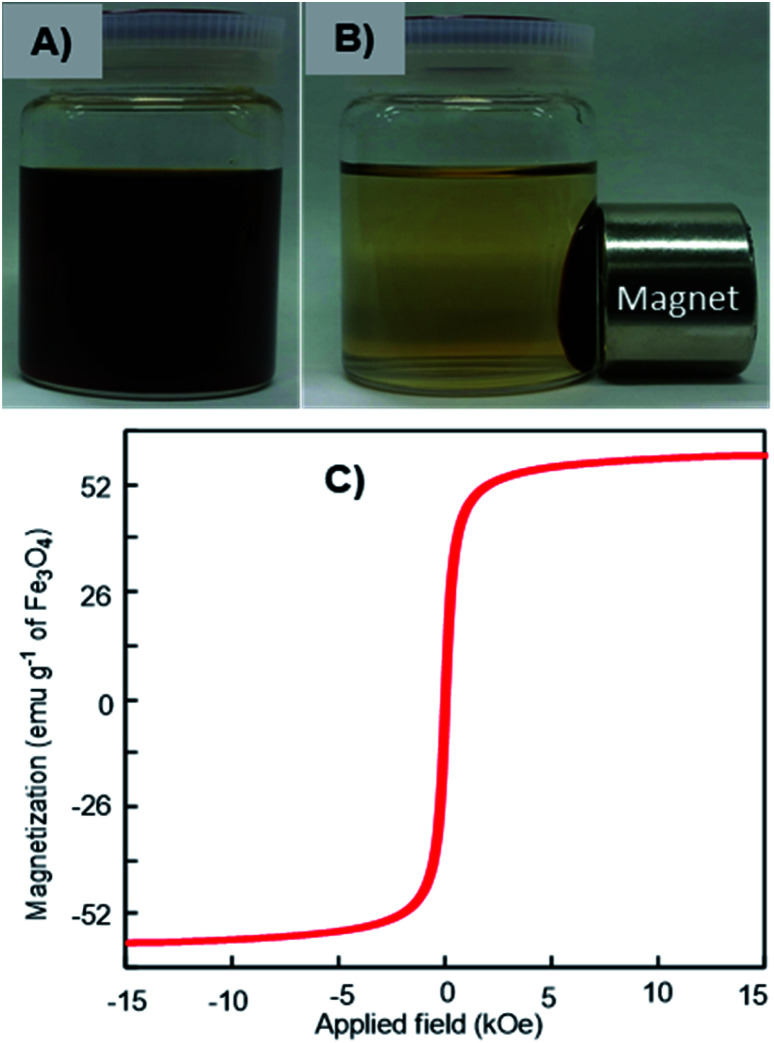
Digital photographs of magnetic separation (A and B) and VSM profile (C) of Fe_3_O_4_-NCCS-FITC-AL nanoparticles at ambient conditions.

The magnetic response of the dispersed Fe_3_O_4_-NCCS-FITC-AL nanoparticles in Dulbecco's phosphate-buffered saline (DPBS) was tested using a permanent magnet. The Fe_3_O_4_-NCCS-FITC-AL nanoparticles are fully dispersible in the absence of external magnetic fields even at the concentration as high as 70 mg mL^−1^ (Fig. 3A). By contrast, in the presence of a strong magnet, the nanoparticles were separated completely within a few minutes (Fig. 3B), while bare Fe_3_O_4_ nanoparticles were instantaneously separated. The excellent dispersity of the Fe_3_O_4_-NCCS-FITC-AL nanoparticles arises from the polyelectrolytic stabilizer, which effectively stabilizes *in situ* synthesized Fe_3_O_4_ while weakens the response towards the external magnetic field. The magnetic property of the charge-inversional Fe_3_O_4_-NCCS-FITC-AL nanoparticles in the dry-state was also analyzed by vibrating sample magnetometry (VSM) at room temperature (Fig. 3C). The *M*–*H* curve of the Fe_3_O_4_-NCCS-FITC-AL nanocomposite particles showed an open hysteresis loop with negligible remanence and coercivity, indicating the superparamagnetic, single domain, and highly crystalline nature of the charge-inversional Fe_3_O_4_-NCCS-FITC-AL nanoparticles. The saturation magnetization (*M*_s_) value of Fe_3_O_4_-NCCS-FITC-AL prepared by the MW treatment is 59 emu g^−1^ in terms of the relative weight composition of Fe_3_O_4_. This value is higher than previously reported Fe_3_O_4_ nanocomposites coated with small molecules, carboxy-CS (42 emu g^−1^)^28^ and a cationic polyelectrolyte (46 emu g^−1^)^48^ prepared without using MW treatment, while it is still lower than that of the typical bulk Fe_3_O_4_ (98 emu g^−1^). The higher *M*_s_ value of the composite particles is ascribable to the presence of the iron phosphonate structures on the surface of the particles, which were secured more stabilized spin contributions than that of the magnetically inactive dead surface layer.^13,14^ The *M*_s_ augmentation has probably originated from the high crystallinity of the Fe_3_O_4_ core. The obtained result validates that the charge-inversional Fe_3_O_4_-NCCS-FITC-AL nanoparticles have enough magnetic efficacy for magnetically-induced manipulation for *in vivo* applications. This MW-assisted synthesis achieves both the excellent magnetic property and the high physiological stability discussed later, which are prerequisites for bio-related applications.

### Optical properties of Fe_3_O_4_-NCCS-FITC-AL nanoparticles

**Fig. 4:**
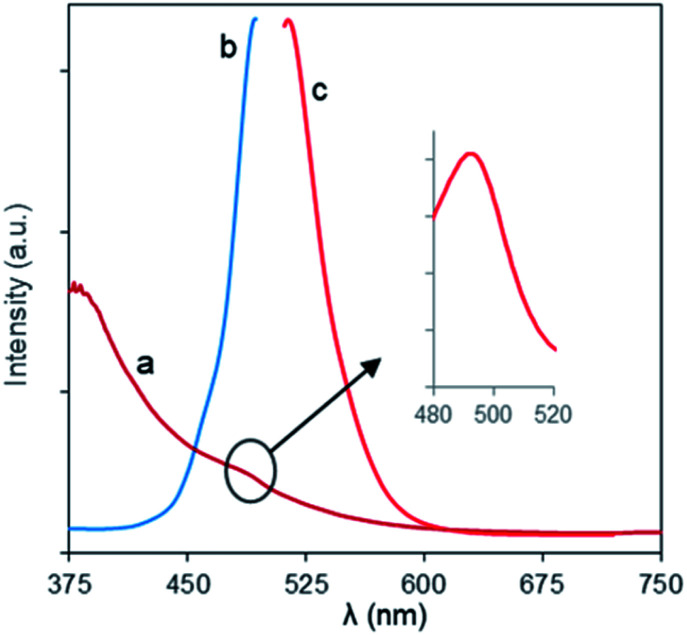
(a) UV-vis, and fluorescence (b) excitation and (c) emission spectra of Fe_3_O_4_-NCCS-FITC-AL nanoparticles in DPBS at ambient conditions (0.30 mg mL^−1^).

The optical behaviors of the charge-inversional Fe_3_O_4_-NCCS-FITC-AL nanoparticles were analyzed by UV-vis absorption, fluorescence excitation and emission spectroscopies (Fig. 4). The polyelectrolytic stabilizer, NCCS-FITC-AL, shows a strong absorption band at 495 nm in the UV-vis spectrum. This clear band originates from the FITC moieties of the stabilizer (Fig. S5†). The UV-vis spectrum of Fe_3_O_4_-NCCS-FITC-AL also showed a shoulder peak around 494 nm for the absorption of Fe_3_O_4_ core, while the intensity of the absorption by the FITC moieties was lower than that of the polyelectrolytic polymer dispersion due to the lower relative content of the FITC moieties in the composite. The emission spectrum of Fe_3_O_4_-NCCS-FITC-AL showed an emission peak with the maximum at 516 nm by excitation at 500 nm. Both the emission and excitation peaks accord with the typical fluorescent behavior of the FITC moieties even in the presence of the carboxy and bisphosphonate moieties and the Fe_3_O_4_ core.^54,55^ The observable fluorescence is sufficient for imaging as described later. This finding agrees well with previous reports.^7,34,54^

### pH-dependent aggregation-dispersion, charge-inversion, and physiological stability of Fe_3_O_4_-NCCS-FITC-AL nanoparticles

**Fig. 5:**
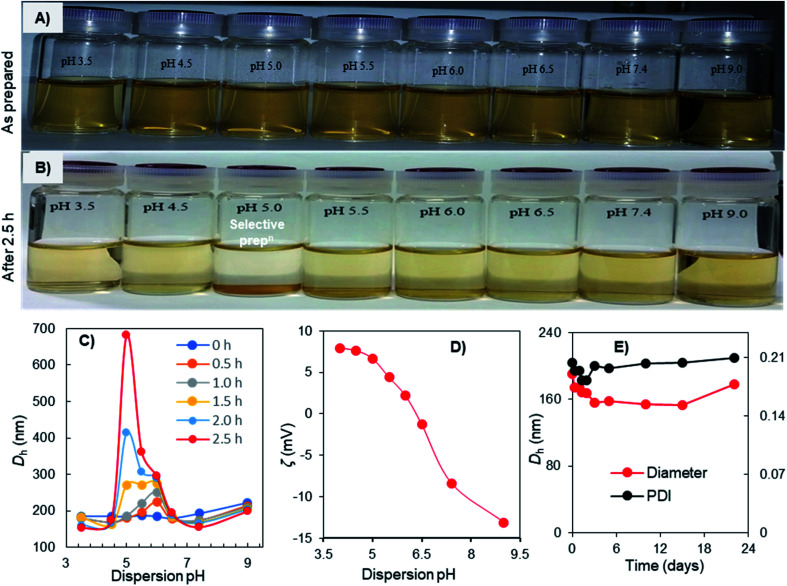
Photo images of pH-dependent aggregation-dispersion (A and B), the pH-dependence of *D*_h_ (C), and ζ-potential (D) of Fe_3_O_4_-NCCS-FITC-AL nanoparticles in DPBS at 25 °C; and time-course of *D*_h_ of Fe_3_O_4_-NCCS-FITC-AL nanoparticles in saline (pH = 7.4) at 25 °C (E).

The aggregation-dispersion and charge-inversional behaviors of the Fe_3_O_4_-NCCS-FITC-AL nanoparticles were examined at a controlled pH range of 3.5–9.0 (Fig. 5). The Fe_3_O_4_-NCCS-FITC-AL nanoparticles were initially well-dispersed in DBPS at the examined pH range (Fig. 5A), while selectively aggregated only at pH 5.0 after 2.5 h (Fig. 5B). This pH responsibility was further studied by DLS. The initial *D*_h_ of Fe_3_O_4_-NCCS-FITC-AL was almost identical (190–200 nm) regardless of the pH values, and the *D*_h_ at pH below 4.5 and above 6.5 was stable. By contrast, the *D*_h_ at the pH range of 5.0–6.0 was gradually increased during the incubation. The increment of *D*_h_ at pH 5.0 was most significant, and the *D*_h_ after 2.5 h reached 690 nm. After that, the nanoparticles were fully settled down and the size exceeded the measurable limit of DLS. This pH-dependent variation of *D*_h_ can be explained by the ζ-potential (Fig. 5C). At the pH range of 3.5–4.5, the Fe_3_O_4_-NCCS-FITC-AL nanoparticles have positive ζ-potentials stabilizing the dispersion by electrostatic repulsions of the ammonium and carboxy groups formed by the protonation of the amine and carboxylate groups. By contrast, at pH 5.0 where the Fe_3_O_4_-NCCS-FITC-AL nanoparticles were selectively precipitated and pH 5.5 and 6.0 where the *D*_h_ was increased, the ζ-potentials became less positive close to zero charge. The precipitation and aggregation of the nanoparticles occurred by the inter-particle attractions among the zwitterionic surface structure. The ζ-potentials were decreased as the increase of the pH values due to the partial deprotonation of the ammonium structure transforming into the amine groups. The weakly positive ζ-potentials in this precipitating region, not isoelectric and higher than those of our previously reported material^56^ implies that a part of the cationic groups does not contribute to the precipitation behavior. A plausible reason is the lower concentration of the free bisphosphoric and carboxyl groups around the surface contributing to the ionic cross-linkage due to the ligation to the Fe_3_O_4_ core as suggested by the FTIR spectroscopic study. Above pH 6.0, the Fe_3_O_4_-NCCS-FITC-AL nanocomposite particles showed negative ζ-potential due to the deprotonation of carboxy and phosphonic groups to form carboxylate and phosphonate groups, respectively, resulting in stable dispersion by the ionic repulsions between the nanoparticles. This pH-dependent charge-conversion property of the nanoparticles having targetable bisphosphonate moieties would be beneficial for selective targeting of acidic bone cells by adhering to the negatively charged cell surface and endocytosis. The physiological stability of the charge-inversional Fe_3_O_4_-NCCS-FITC-AL nanoparticles dispersed in saline was confirmed by studying their *D*_h_ and PDI at physiological conditions (pH 7.4, 37 °C) for 3 weeks (Fig. 5E). Both the *D*_h_ (∼200 nm) and PDI (below 0.20) of the particles were not significantly changed throughout the observation period because of the electrostatic repulsions among the negatively charged carboxylate and phosphonate moieties of the polyelectrolytes. This result demonstrates that this magnetic and fluorescent nanosystem has capabilities of prolonged circulation and selective deposition in locally acidic environments.

### Hemocompatibility of Fe_3_O_4_-NCCS-FITC-AL nanoparticles

**Fig. 6:**
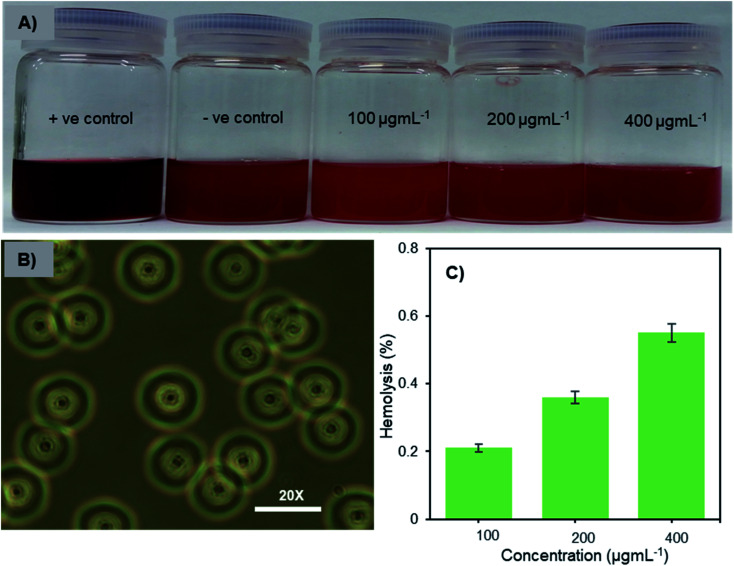
Optical images of sheep erythrocytes dispersed in saline containing Fe_3_O_4_-NCCS-FITC-AL nanoparticles (A), optical microscopic photograph of sheep erythrocytes dispersed in saline containing 400 μg mL^−1^ Fe_3_O_4_-NCCS-FITC-AL nanoparticle (B), and concentration dependence of hemolysis of sheep erythrocytes dispersed in saline with Fe_3_O_4_-NCCS-FITC-AL (C) (concentration of erythrocyte = 200 μL mL^−1^, pH = 7.4, incubation time = 5 h, 37 °C).

Hemocompatibility is one of the important prerequisites of biomaterials for intravenous applications. The concentration-dependent hemolytic behaviour of Fe_3_O_4_-NCCS-FITC-AL was examined using sheep erythrocytes in saline at pH 7.4 and 37 °C for 5 h. Sheep erythrocytes dispersed in a 1% aqueous solution of Triton X 100 and saline were employed as the positive and negative controls, respectively (Fig. 6A). The treatment with Triton X-100 completely hemolyzed the erythrocytes through membrane disruption, and the color was turned into dark red, while the red color of the erythrocytes was retained in the negative control sample. The colors of the erythrocyte suspensions containing Fe_3_O_4_-NCCS-FITC-AL gradually became slightly darker by trace degrees of hemolysis enhanced as the increase of the concentration of Fe_3_O_4_-NCCS-FITC-AL (Fig. 6A). At pH 7.4, Fe_3_O_4_-NCCS-FITC-AL nanoparticles showed only 0.21% hemolysis at 100 μg mL^−1^, while they showed hemolysis approximately three times higher at 400 μg mL^−1^ (Fig. 6C). The hemolysis degrees of Fe_3_O_4_-NCCS-FITC-AL are significantly lower than that of previously reported magnetic nanocomposite particles coated with cationic polyelectrolyte,^48^ carboxy-CS,^28^ and functionalized carboxy-CS.^56^ The morphological change of the sheep erythrocytes in the presence and absence of Fe_3_O_4_-NCCS-FITC-AL was confirmed by optical microscopy (Fig. 6B). Most of the erythrocytes incubated with Fe_3_O_4_-NCCS-FITC-AL retained their native morphologies in the same manner with the negative control (Fig. S8†). The improved hemocompatibility of Fe_3_O_4_-NCCS-FITC-AL plausibly originates from the weakly negative surface charge, which is smaller than natural erythrocytes, resulting in negligible interactions with the negative membrane surface of erythrocytes under these conditions. The hemocompatibility of Fe_3_O_4_-NCCS-FITC-AL meets the acceptable limit of blood-contacting biomaterials (5%) under the physiological conditions,^57,58^ indicating the potential of Fe_3_O_4_-NCCS-FITC-AL for intravenous applications.

### pH-triggered membrane disruption of erythrocytes by Fe_3_O_4_-NCCS-FITC-AL nanoparticles

**Fig. 7:**
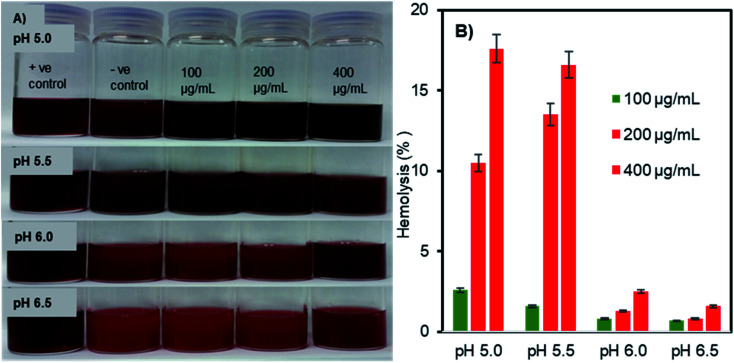
(A) Optical images, and (B) hemolysis degrees of pH- and concentration-dependent membranolysis of sheep erythrocytes by charge-inversional Fe_3_O_4_-NCCS-FITC-AL nanoparticles (in saline, incubation time = 5 h, 37 °C, concentrations of Fe_3_O_4_-NCCS-FITC-AL = 100, 200, and 400 μg mL^−1^).

The pH- and concentration-dependent membrane disruption abilities of the charge-inversional Fe_3_O_4_-NCCS-FITC-AL nanoparticles were tested by hemolysis assay using sheep erythrocytes as a model cell (Fig. 7). The optical images (Fig. 7A) show the relative colour changes of the erythrocytes dispersion with the variations of pHs and concentrations of Fe_3_O_4_-NCCS-FITC-AL. Erythrocytes dispersed in 1% Triton X100, which fully lysed the erythrocytes was used as positive controle. While erythrocytes dispersed in saline having minimal lysis was regarded as negative controle. The red coloured-dispersion of Fe_3_O_4_-NCCS-FITC-AL gradually turned into black with decreasing pHs and increasing nanoparticle concentrations. The hemolysis degree of the erythrocytes by the Fe_3_O_4_-NCCS-FITC-AL nanoparticles was increased with the decrease of pH. This enhancement of hemolysis is ascribable to the decrease in the negative surface charge of the nanoparticles that lead to enhanced adherence to the erythrocytes membrane surface. The hemolysis degree became significantly higher (18.7%) at the pH range of 5.5 to 5.0, where the negative surface charge of the Fe_3_O_4_-NCCS-FITC-AL nanocomposite particles changes to positive (Fig. 7B). The positive charge originates from the protonation of the acid-sensing secondary amine, carboxy, and phosphonate groups, enhancing electrostatic interactions with the negatively charged lipid membrane of the erythrocytes under the acidic environments. Another plausible reason for the increase of the hemolysis degree is the hydrophobic interactions between the pendant hydrophobic structures in the polyelectrolyte coating with the lipid membrane of the erythrocytes. At pH 5.0 in the presence of the charge-inversional Fe_3_O_4_-NCCS-FITC-AL nanoparticles, the erythrocyte membranes were significantly damaged and the erythrocytes were obviously agglutinated (Fig. S9†). This pH-sensible membrane disruption suggests the acidity-responsible membranolytic ability of Fe_3_O_4_-NCCS-FITC-AL nanoparticles at the pH range from 5.0 to 5.5 identical to the endosomal pH. Similar behaviour was also reported for MIONPs for selective disruption of cancer cells only under the acidic pH.^58,59^ Fe_3_O_4_-NCCS-FITC-AL meets a requirement for intravenous applications that nanoparticles spontaneously become membranolytic under the acidic conditions with high hemocompatibility under the physiological conditions.

**Fig. 8:**
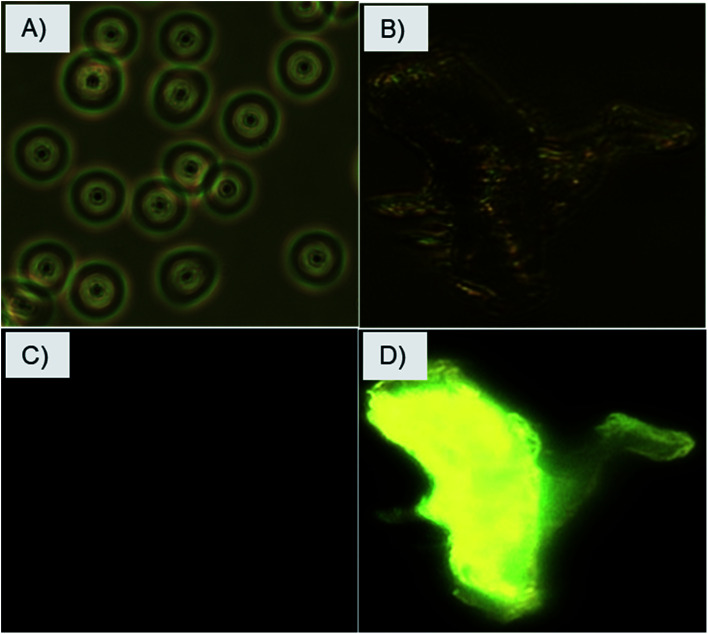
Optical and fluorescence microscopic images of erythrocytes incubated with charge-inversional Fe_3_O_4_-NCCS-FITC-AL nanocomposite particles at pH 7.4 (A, optical image; C, fluorescence image) and at pH 5.0 (B, optical image; D, fluorescence image).

The selective interactions and adherence of the charge-inversional Fe_3_O_4_-NCCS-FITC-AL nanoparticles to the erythrocyte membranes were further confirmed by fluorescence microscopy (Fig. 8). At pH 7.4, the erythrocytes show their regular biconcave circular shape in the light microscopic image (Fig. 8A), and fluorescent parts are invisible in the fluorescent microscopic image of the erythrocytes (Fig. 8C). These images indicate that Fe_3_O_4_-NCCS-FITC-AL charged negatively under physiological conditions without interaction with the erythrocytes. By contrast, the erythrocyte membranes were significantly broken at pH 5.0 due to the strong adherence of positively charged Fe_3_O_4_-NCCS-FITC-AL on the erythrocyte membranes (Fig. 8B). The accumulation of Fe_3_O_4_-NCCS-FITC-AL on the erythrocyte membranes was clearly confirmed by the fluorescent part in the fluorescence image matching with the morphology observed in the optical image (Fig. 8D). This observation implies the practical application of Fe_3_O_4_-NCCS-FITC-AL for fluorescence imaging visualizing cellular interactions.

### pH-dependent bone mineral affinity of Fe_3_O_4_-NCCS-FITC-AL

**Fig. 9:**
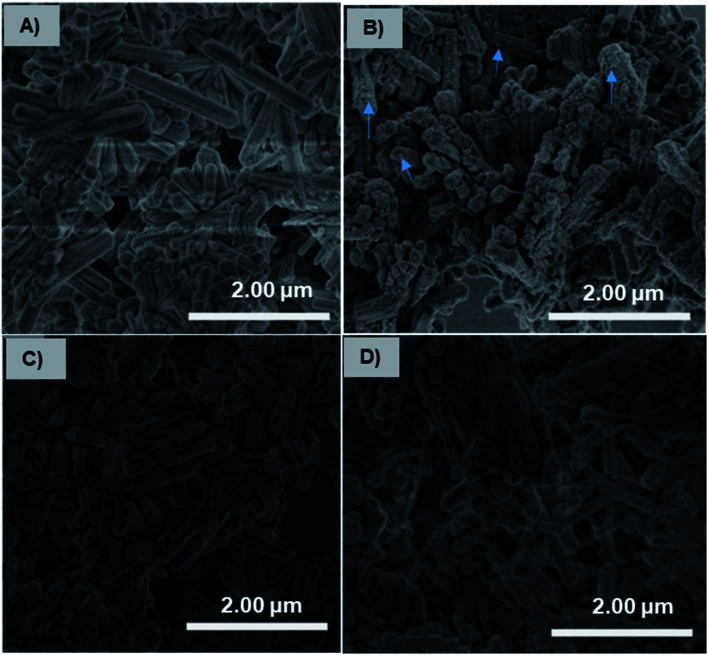
SEM images of pristine HAp (A and C) and HAp treated with Fe_3_O_4_-NCCS-FITC-AL (B and D) at pH 5.0 (A and B) and 7.4 (C and D) (saline, incubation time = 3 h, 37 °C, 50 μg mL^−1^ of Fe_3_O_4_-NCCS-FITC-AL). Blue arrows indicate the adherence of Fe_3_O_4_-NCCS-FITC-AL.

The charge-inversional Fe_3_O_4_-NCCS-FITC-AL nanoparticles were designed for bone-seeking diagnosis and therapeutic applications. Therefore, we investigated the pH-dependent affinity of Fe_3_O_4_-NCCS-FITC-AL towards bone minerals. HAp with a monoclinic structure having 2.5 μm of the average length was used as a model bone mineral. HAp has the chemical formula of 3Ca_3_(PO_4_)_2_·Ca(OH)_2_, and its surface composes of Ca^2+^, OH^−^, and PO_4_^3−^ ions with the protonated phosphate ions depending on pH and coexisting ions.^61^ HAp and Fe_3_O_4_-NCCS-FITC-AL were incubated at 37 °C in saline and the dispersion pH was adjusted to acidic (5.0) and physiological (7.4) conditions. The adhesion ability of the Fe_3_O_4_-NCCS-FITC-AL nanoparticles to HAp surface was studied by SEM and EDX analyses (Fig. 9). Blank experiments without Fe_3_O_4_-NCCS-FITC-AL were also performed at both pHs of 5 and 7.4 to observe the effects of the acidic environment (H^+^ rich) and saline (Na^+^ and Cl^−^ ions) on the surface of HAp. Any changes caused by these ions were not observable for both of the control samples (Fig. 9A and C). In addition, adhered particles were not found in the SEM image of HAp treated with Fe_3_O_4_-NCCS-FITC-AL under the physiological conditions (Fig. 9D), which indicates the very poor affinity to HAp. This low affinity is plausibly due to the ionic repulsions between the negative HPO_4_^2−^ moieties on the surface of HAp and the negatively charged Fe_3_O_4_-NCCS-FITC-AL under physiological conditions.^60,61^ The content of iron on the treated HAp surface was calculated to be below 0.01 wt% from EDS. The SEM image of HAp treated with Fe_3_O_4_-NCCS-FITC-AL under the acidic pH (5.0) shows many adhered particles indicated by blue arrows (Fig. 9B). The existence of Fe_3_O_4_-NCCS-FITC-AL was also confirmed by EDS analysis, and the Fe content on the surface of HAp was calculated to be 1.4 wt%. The increased affinity of Fe_3_O_4_-NCCS-FITC-AL toward HAp can be explained by the strong electrostatic interactions and hydrogen bonding. The surface of HAp under acidic conditions is covered by H_2_PO_4_^−^ groups, in which the P=O remains facing-up and Ca^2+^ ions are unveiled by the dissolution of anions.^60–62^ The Ca^2+^ ions on the HAp surface electrostatically attract the carboxylate and bisphosphonate moieties of the Fe_3_O_4_-NCCS-FITC-AL nanocomposite particles. Similar electrostatic interactions were also observed in the previously reported materials bearing bisphosphonate moieties.^61,63–65^ In addition, the ammonium and P–OH groups present on the surface of the charge-inverted Fe_3_O_4_-NCCS-FITC-AL nanocomposite particles also contributed to the attraction by the electrostatic and hydrogen-bonding interactions with the surface groups like H_2_PO_4_^−^, PO_4_^3−^ and P=O moieties of HAp.^3,15^

**Fig. 10:**
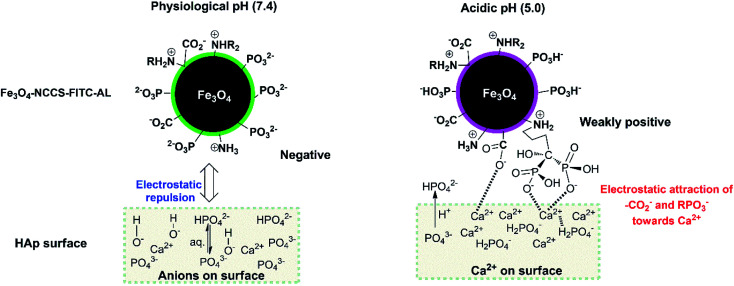
Schematic representation of the bindings of Fe_3_O_4_-NCCS-FITC-AL nanoparticles to HAp.

This stronger adherence under the acidic conditions, approximately 140% higher affinity than that under the physiological conditions (Fig. S10†), is plausibly ascribed to both the physisorption and chemisorption of the nanoparticles on the HAp surface (Fig. 10). The surface of Fe_3_O_4_-NCCS-FITC-AL is covered by the anionic bisphosphonate and carboxylate ions and cationic quaternary ammonium ions that electrostatically interact with Ca^2+^ and H_2_PO_4_^−^ on the surface of HAp, respectively, even though the zeta potential is not so high due the buffering ability of the polyampholytic skeleton. In addition, the dissolution–deposition equilibrium of the phosphonate ions of HAp results in the incorporation of the bisphosphonate groups of Fe_3_O_4_-NCCS-FITC-AL into the lattice structure of HAp (Fig. 10, right part). These interactions of phosphonates with HAp were reported in previous NMR spectroscopic (^31^P, ^15^N, ^13^C, ^2^H, and ^1^H) and chromatographic studies.^61,63,66,67^ This finding revealed that the designed nanosystem has adequate potentials for selective targeting of acidic bone sites.

### Affinity of Fe_3_O_4_-NCCS-FITC-AL nanoparticles towards native bone sample

**Fig. 11:**
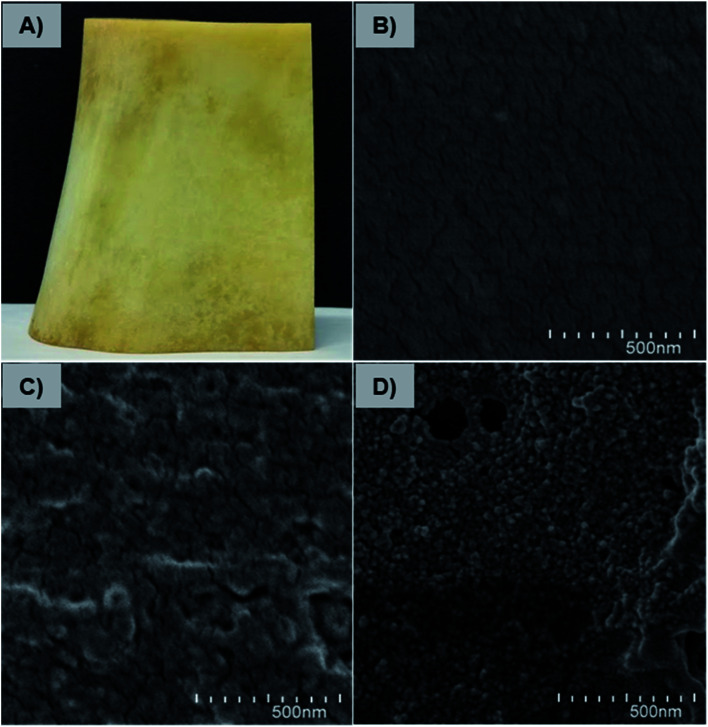
Optical (A) and SEM (B) images of pristine bovine bone, and SEM images bovine bone treated with charge-inversional Fe_3_O_4_-NCCS-FITC-AL nanocomposite particles at pH 7.4 (C), and at pH 5.0 (D) (incubation time, 5 h, 37 °C, 50 μg mL^−1^ of Fe_3_O_4_-NCCS-FITC-AL).

The affinity and adherence of the charge-inversional Fe_3_O_4_-NCCS-FITC-AL nanoparticles towards native bone samples were studied by SEM analysis. Bovine leg bone samples were treated with Fe_3_O_4_-NCCS-FITC-AL at pH 7.4 and 5.0 (Fig. 11). The optical and SEM images of the bone sample are shown in Fig. 11A and B, respectively. Both the optical and SEM images of the reference bone showed smooth surface morphology and the absence of any deposited materials except some cracks. The SEM image of the bone sample incubated with Fe_3_O_4_-NCCS-FITC-AL nanoparticles at pH 7.4 showed almost identical morphology, and Fe_3_O_4_-NCCS-FITC-AL adhered was not observable (Fig. 11C). The poor affinity is due to the electrostatic repulsions between the negatively charged Fe_3_O_4_-NCCS-FITC-AL nanoparticles and the bone surface under the physiological conditions. By contrast, at pH 5.0, Fe_3_O_4_-NCCS-FITC-AL nanoparticles were adhered on the bone surface, which was almost covered by these nanoparticles (Fig. 11D). This pH dependence, namely high affinity only under acidic conditions, is identical to HAp employed as the model system, while the enhancement in the affinity to the bone under the acidic conditions is more significant than that to HAp. Bone mainly consists of HAp and collagen, whose solubility is higher at pH 5.0 by the positively charged nature.^42,68^ The increase in the active surface of HAp by the dissolution of charged collagen is a plausible factor for higher affinity. In metastasized bone sites, degradation of collagen is enzymatically promoted, and this acidity-sensitive adsorption is presumably more enhanced. Osteoclasts become acitivated by cancers and overexpress cathepsin-K degrading collagen fibers networks.^5^ This synergy of the pH-triggered charge-conversion and targetable bisphosphonate moieties in the Fe_3_O_4_-NCCS-FITC-AL nanoparticles enabled selective accumulation on HAp unvailed under acidic conditions.

## Conclusions

In this report, we demonstrated a magnetic nanomaterial, Fe_3_O_4_-NCCS-FITC-AL having pH-controlled hemocompatibility and bone-targeting ability. Fe_3_O_4_-NCCS-FITC-AL was prepared by a simple one-step aqueous process called the *in situ* coprecipitation-coating method without requiring any tedious post modification, toxic organic solvents, capping ligand, stabilizer, and emulsifier. Fe_3_O_4_-NCCS-FITC-AL consists of spherical cores of Fe_3_O_4_ with sizes around 10 nm, and the *D*_h_ of 160 nm under physiological conditions suits well for circulation. Fe_3_O_4_-NCCS-FITC-AL is paramagnetic and magnetically guidable. While Fe_3_O_4_-NCCS-FITC-AL is highly dispersible under physiological conditions, it is selectively precipitated at pH 5.0. This pH-dependent change in the dispersibility originates from the charge conversion of the acidic moieties. The hemolytic assay demonstrated excellent hemocompatibility of Fe_3_O_4_-NCCS-FITC-AL at physiological conditions due to the presence negatively charged biocompatible coating. But, Fe_3_O_4_-NCCS-FITC-AL becomes highly hemolytic under acidic conditions owing to the charge reversion ability of the polyelectrolyte. Fluorescent imaging confirmed adherence of Fe_3_O_4_-NCCS-FITC-AL to the membranes of erythrocytes only under acidic conditions. The interactions of Fe_3_O_4_-NCCS-FITC-AL particles with a bone sample and HAp changed with pH-variations in a similar manner with the hemolytic behavior. At pH 7.4, the negatively charged Fe_3_O_4_-NCCS-FITC-AL particles did not interact with HAp and bone sample due to the strong electrostatic repulsions, suggesting the minimal systemic and off-targeted side effects. At acidic pH (5.0), the charge-inverted Fe_3_O_4_-NCCS-FITC-AL was adhered on HAp and the bone sample by the bone-seeking bisphosphonate moieties and the electrostatic interactions, implying the possibility of the selective targeting of malignant bone tissues. Therefore, designed Fe_3_O_4_-NCCS-FITC-AL particle is a promising candidate for early-stage diagnosis and therapy of bone malignancies.

## Experimental section

### Material and methods

#### Materials

CS (*M*_w_ = 5.6 × 10^5^ g mol^−1^), CAn, and AL were purchased from Tokyo Chemical Industry (Tokyo, Japan). FeSO_4_·7H_2_O and FITC were purchased from Sigma Aldrich (St. Louis, MO, USA). NH_4_OH (28%) and FeCl_3_·6H_2_O were purchased from Kanto Chemical (Tokyo, Japan). HAp was purchased from Wako Pure Chemical (Osaka, Japan). DPBS was purchased from Gibco Life Technologies (Paisley, UK). Preserved sheep whole blood was purchased from Cosmo Bio (Tokyo, Japan). Bovine leg bone was purchased from Baticrom Halal Shop (Tokyo, Japan). All other reagents used in this study were lab grade and used without further purification. Water was purified using a Nomura Micro Science (Kanagawa, Japan) MINIPURE TW-300RU water purification system. Deionized distilled water (DDW) was used throughout the study.

#### Measurements

FTIR spectra were documented on a JASCO (Tokyo, Japan) FTIR-460 plus spectrometer using pressed KBr pellets with a scan rate of 4 cm^−1^sec^−1^. ^1^H NMR spectra were recorded on a JEOL (Tokyo, Japan) ECX-400 instrument using tetramethylsilane as an internal standard (400 MHz) at room temperature. Hydrodynamic diameters were measured by DLS on a Malvern Instrument (Malvern, UK) Zetasizer Nano ZS. The size of dried nanoparticles was measured using SEM images taken with a Hitachi (Tokyo Japan) SU8000 microscope operated at an accelerating voltage of 30 kV. The powder X-ray diffraction (XRD) patterns were measured on a Rigaku (Tokyo, Japan) Ultima IV RINT D/max-kA diffractometer with Cu Kα radiation (*λ* = 1.54178 Å). TEM images were taken using a JEOL (Tokyo, Japan) TEM-2100F field emission scanning electron microscope. Thermogravimetric analysis was carried out on a Seiko Instrument (Tokyo, Japan) TG/DTA 6200 (EXSTER6000) at a heating rate of 10 °C min^−1^ under N_2_. The magnetic properties of the nanoparticles were analyzed on a Riken Denshi (Tokyo, Japan) BHV-30 series vibrating sample magnetometer at ambient conditions. Energy-dispersive X-ray spectra were recorded on a JEOL (Tokyo, Japan) JSM-6510A analytical scanning electron microscope. The optical absorbance was taken using an AS ONE ASV11D UV-visible spectrophotometer. The erythrocytes specimens were observed with an Olympus (Tokyo, Japan) CKX53 microscope. The color pictures of the specimens were captured using a Visualix Pro2 (Kobe, Japan) camera. All images were taken at the same magnification (20×).

### Methods

#### Synthesis of bisphosphonated polyelectrolytic CS derivative: *N*-carboxycitraconylation of CS (NCCS)

CS was dissolved in 200 mL of aqueous acetate buffer (pH 4.6) by magnetic stirring overnight. The solution was adjusted at pH 5.6 using dilute aqueous NaOH solution. CAn was diluted in methanol (10 mL) and dropwise added to CS solution for 1 h. The reaction was continued for 12 h at room temperature. After the reaction, 10 M NaOH aqueous solution was added to the reaction mixture and stirred for an additional 10 min. The reaction mixture was added dropwise to an excess amount of acetone to precipitate NCCS. The precipitate was separated using a Buchner funnel. The resulting product was purified *via* dispersing in DDW and reprecipitated in acetone. The purified NCCS was dried in a vacuum desiccator and stored in a refrigerator.

#### FITC labeling of NCCS

NCCS (800 mg) was dissolved in DDW (300 mL). FITC (4.00 mg) was dissolved in 10 mL methanol and the solution was added dropwise to the mixture for 1 h. The reaction was continued in dark conditions for 4 h at room temperature. The reaction mixture was added dropwise to an excess amount of methanol to precipitate the NCCS-FITC. The resulting product was separated by centrifugation at 4000 rpm and washed repeatedly until the decant became completely colorless. The obtained NCCS-FITC was dried in a desiccator in dark and stored in a freezer at −4 °C.

#### Bisphosphonylation of NCCS-FITC

In dark conditions, NCCS-FITC was dissolved in DDW (200 mL) by magnetic stirring at room temperature. AL (100 mg) was dissolved in 10 mL DDW and the solution was added dropwise to the dispersion of NCCS-FITC. The reaction was performed at room temperature in dark conditions for 24 h. After the reaction period, the product was precipitated by pouring the mixture into an excess amount of acetone and separated by filtration. The resulting product was purified by dispersing in DDW and reprecipitating in methanol. Bisphosphonated polyelectrolytic NCCS-FITC-AL was obtained by drying in a vacuum desiccator (yield = 85%) and kept at −15 °C.

#### MW-assisted *in situ* synthesis and surface functionalization of Fe_3_O_4_ nanoparticles

Bisphosphonated polyelectrolytic NCCS-FITC-AL was dissolved in deoxygenated DDW (175 mL) by magnetic stirring. FeCl_3_·6H_2_O (270 mg, 1.00 mmol) and FeSO_4_·7H_2_O (139 mg, 0.50 mmol) were dissolved in 15 mL DDW with purging N_2_. These solutions were mixed in a three-necked round bottom flask (300 mL) under N_2_. The flask was placed in a preheated thermostat oil bath maintained at 80 °C. Aqueous ammonia solution (5 mL, 28%) was injected at a rate of 1 mL min^−1^. The mixture was magnetically stirred for 10 min. The reaction mixture was, then, quickly transferred into a 500 mL conical flask. The flask was placed in a MW oven to be heated at 100 °C for 30 min. The resultant black precipitate was magnetically washed repeatedly with DDW until the supernatant became neutral pH. The solid precipitate was separated at the bottom of the flask and dried in a vacuum desiccator for 24 h. Bare Fe_3_O_4_ was synthesized following the aforementioned procedure without adding the stabilizer, NCCS-FITC-AL.

#### Hemocompatibility of Fe_3_O_4_-NCCS-FITC-AL

Hemolysis activities of the Fe_3_O_4_-NCCS-FITC-AL nanoparticles were assayed by using the following protocol. Preserved sheep whole blood (40 mL) was centrifuged at 2500 rpm for 5 min. The sheep erythrocytes were separated by centrifugation and plasma removal and washed three times with saline (150 mM NaCl aq.). The washed erythrocytes were redispersed in saline (40 mL). The nanoparticles were also dispersed in saline and the dispersion was mixed with erythrocytes suspension. The pH of the mixtures was adjusted to 7.4 using 0.01 M HCl aq. The final concentrations of the Fe_3_O_4_-NCCS-FITC-AL nanoparticles were adjusted to 100, 200, and 400 μg mL^−1^. Triton X-100 (1% aqueous solution) and saline were added to the erythrocytes suspension to prepare the positive (100% lysis) and negative (0% lysis) control samples, respectively. All the samples and controls were incubated for 5 h at 37 °C. The samples were gently swirled once per 20 min for resuspending the erythrocytes. After incubation, the suspension was centrifuged at 10 000 rpm for 5 min to separate the erythrocytes and the supernatants were incubated for 30 min at room temperature to oxidize the released hemoglobin. The optical absorbance of oxyhemoglobin was assessed by absorption at 540 nm. The hemolysis percentage of erythrocytes was calculated using the following equation:% hemolysis = {(Abs_sample_ − Abs_negative control_)/(Abs_positive control_ − Abs_negative control_)} × 100
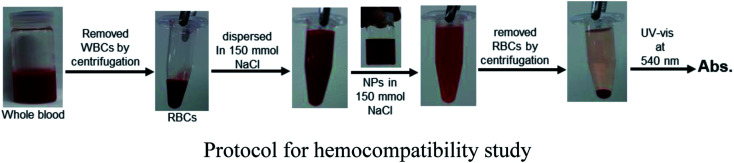


#### Membrane morphology study of erythrocytes

The morphology of erythrocytes was studied using a light microscope. The erythrocytes were added to saline containing the charge-conversional Fe_3_O_4_-NCCS-FITC-AL nanoparticles at 400 μg mL^−1^, and the pH values were set to 7.4 and 5.0 using 0.01 M HCl aqueous solution. The resulting dispersions were incubated at 37 °C for 5 h, and mildly shaken once every 20 min. After the incubation period, the dispersions were treated with a magnet to remove free nanoparticles. Then, the erythrocytes were cleaned by centrifugal washing and resuspended in saline. A control sample was also made without using nanoparticles. The washed erythrocytes suspension (10 μL) was placed on a glass slide and covered with a coverslip glass. The resulting specimens were observed microscopically.

#### pH-selective adhesion of Fe_3_O_4_-NCCS-FITC-AL to HAp and native bone sample

Fe_3_O_4_-NCCS-FITC-AL nanoparticles (500 μL) were dispersed in saline (10 mL) and the pH values of the dispersions were adjusted to 7.4 and 5.0 using 0.01 M HCl aqueous solution. HAp (20 mg) was added to each dispersion and dispersed by mild shaking. The dispersions were incubated at 37 °C for 3 h and shaken once every 20 min for redispersion. After incubation, the HAp particles were separated by centrifugation at 2000 rpm and washed 7 times with saline. Finally, the washed HAp was analyzed by SEM. For the native bone sample, a small piece of clean bone was added to the dispersion instead of HAp, incubated under the aforementioned conditions, and washed 7 times with saline and water to remove the free and loosely bound nanoparticles. The control bone samples were made without using Fe_3_O_4_-NCCS-FITC-AL nanoparticles. All the bone samples were also analyzed by SEM and EDS.

## Author contributions

M. A. Rahman conceptualized the idea and did formal experiments. He collected and analysed the data, and also explained the results. This research was supervised by B. Ochiai. The manuscript was drafted by M. A. Rahman and revised by B. Ochiai. The final version of the manuscript has approved by both the authors.

## Conflicts of interest

The authors declare no competing financial interests that would have been arised to influence the work reported in this paper.

## Supplementary Material

RA-012-D1RA09445A-s001
