# Supplementary material for: A facile aqueous production of bisphosphonated-polyelectrolyte functionalized magnetite nanoparticles for pH-specific targeting of acidic-bone cells
Source: RSC Adv. 2022 Mar 11;12(13):8043–58. doi: 10.1039/d1ra09445a (PMC8982438; doi:10.1039/d1ra09445a)
Supplement: RA-012-D1RA09445A-s001 [file RA-012-D1RA09445A-s001.pdf]

Electronic Supplementary Material (ESI) for RSC Advances.
This journal is © The Royal Society of Chemistry 2022

A facile aqueous production of bisphosphonated-polyelectrolyte functionalized magnetite nanoparticles for pH-specific targeting of acidic-bone cells

Md. Abdur Rahman[1,2], Bungo Ochiai[1,*]

[1] Department of Chemistry and Chemical Engineering, Graduate School of Science and Engineering, Yamagata University, 4-3-16, Jonan, Yonezawa, Yamagata 992-8510, Japan.

[2] Polymer Colloids and Nanomaterials Lab, Department of Chemistry, Faculty of Science, Rajshahi University, Rajshahi-6205, Bangladesh.

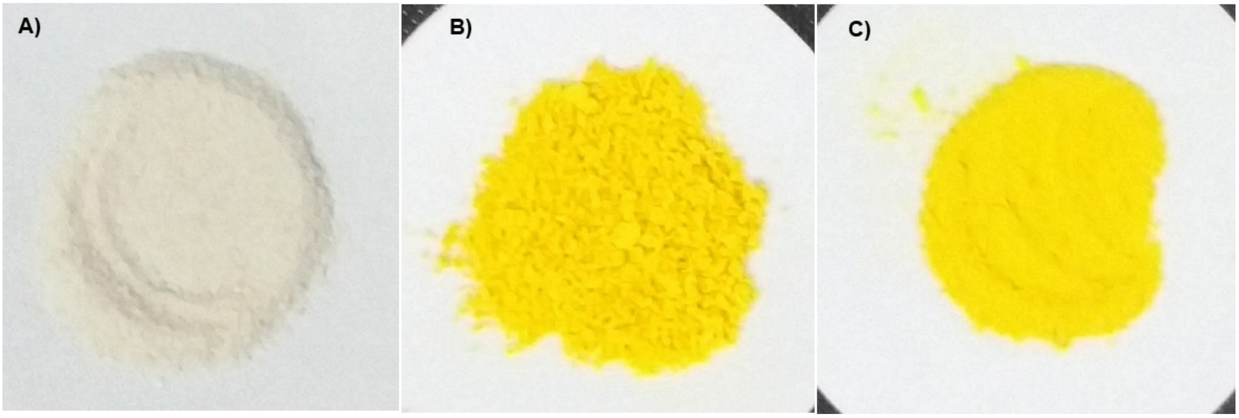

Figure S1: Optical images of (A) *N*-citraconyl chitosan (NCCS), (B) NCCS-fluorescein isothiocyanate (NCCS-FITC), and (C) NCCS-FITC-alandronate (NCCS-FITC-AL).

Fig. S2 shows the EDX spectra of unmodified CS, NCCS, NCCS-FITC, and polyelectrolytic NCCS-FITC-AL. The spectrum of CS showed only the signals for C, N, and O. The presence of a new peak of Na in the spectrum of NCCS certifies the modification of CS with citraconic anhydride (CAn) followed by neutralization with NaOH aq. Although, the FITC labelling of NCCS was significantly changed the colour of product, the overall elemental composition in the EDS spectrum of NCCS-FITC was not altered due to the trace sulfur content. The existence of a new and significant peak for P-atom in the EDX spectrum of NCCS-FITC-AL also supports the bisphosphonylation of NCCS-FITC.

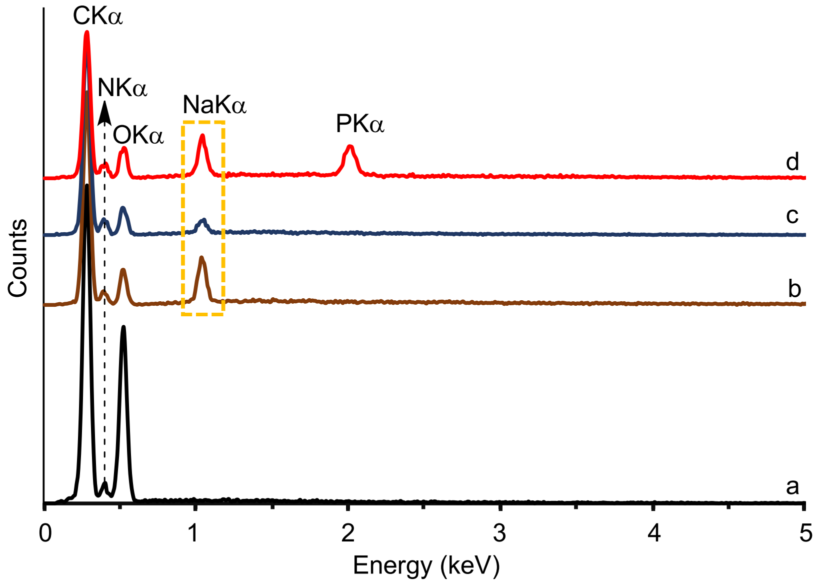

Figure S2: EDX spectra of (a) CS, (b) NCCS, (c) NCCS-FITC, and (d) NCCS-FITC-AL.

Fig. S3 illustrates the FTIR spectra of CS, NCCS, NCCS-FITC, and NCCS-FITC-AL. The characteristic absorption of CS are those observed at 3417 cm^{-1} assignable for O–H and N–H stretching, 2920 and 2873 cm^{-1} for C–H asymmetric and symmetric stretching, 1652 cm^{-1} for amide-I stretching with O–H, N–H and C–H bending, 1558 cm^{-1} for amide–II stretching, 1376 cm^{-1} for C–N stretching, 1151 cm^{-1} for C–O–C bridge of saccharide unit, 1074 cm^{-1} for C–O stretching.[1,2] In addition to these peaks of CS, the FTIR spectrum of NCCS shows characteristic bands at 1406 cm^{-1} assignable to the carbonyl stretching of carboxylate ions[3] of the Na-citraconyl group, 1636 cm^{-1} assignable to the C=C stretching, and 3086 cm^{-1} for =C–H stretching. In addition, the widths and intensities of the O–H stretching, and amide–II stretching bands were significantly changed due to the improvement in hydrophilic characteristic of NCCS via amidation of the primary amine moieties of CS with CAn. The reported characteristic FTIR peaks of FITC are those at 1458, 1535, and 1594 cm^{-1} assigned to the aromatic ring stretching and at 2015 cm^{-1} assigned to the stretching of N=C=S vibration band.[4] The FTIR spectrum of NCCS-FITC also shows the corresponding peaks at 1448 and 1559 cm^{-1}, while the peak at 1594 cm^{-1} is invisible due to overlapping with other absorption. In addition, no observable peak was appeared at 2015 cm^{-1}, which indicates that the FITC group observed is the introduced one and not originated from unreacted FITC. The FTIR spectrum of NCCS-FITC-AL shows characteristic peaks assignable to the bisphosphonate moieties. The peaks at 1036 cm^{-1} assignable to P–O stretching and at 1108

cm^{-1} for P=O stretching are observed as the reported spectrum of AL.[5,6] In addition, minor changes were observable at the C=C and =C–H peak positions due to the addition of -NH_2 groups to the alkenyl moieties.

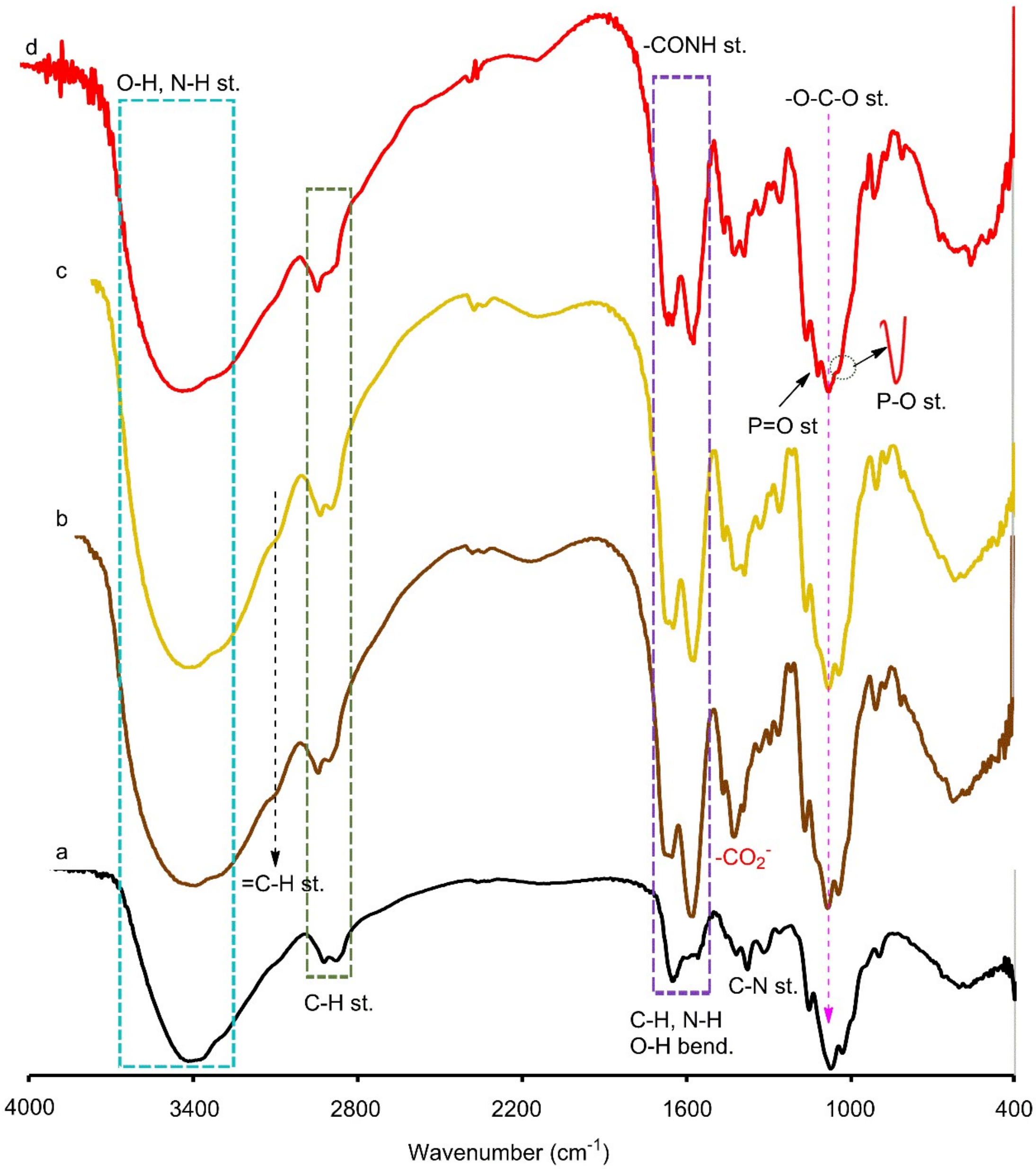

Figure S3: FTIR spectra of (a) CS, (b) NCCS, (c) NCCS-FITC, and (d) NCCS-FITC-AL.

The ^{1}H NMR spectrum of CS shows characteristic signals at 2.3 ppm assignable to CH_3 protons in the acetyl groups, 2.9 ppm for C_2H proton, 3.3-3.9 ppm assignable to the protons of the saccharide rings (Fig. S4). The C_1H proton was observed at 5.3 ppm. In the spectrum of NCCS, some characteristic new peaks are observed. The signal at 1.8 ppm is assignable to the -NHCOCH=CCH_3COONa protons originating from the citraconyl moieties. The characteristic two new signals appeared at 5.4 and 5.8 ppm are assignable to the alkenyl protons in the proximal and distal isomers, respectively, produced via the addition of CAn to the primary amine moieties of CS, which are in accordance with the previous report [7]. The signals of aromatic protons originating from the FITC residue are not detectable in the ^{1}H NMR spectrum of NCCS-FITC due to the presence of very trace amount of the introduced structure[8] to the NCCS chain. The spectrum of NCCS-FITC-AL shows a new peak at 2.9 ppm assignable to the CH_2 protons originating from the addition of bisphosphonate, AL to the alkenyl groups. In addition, the peak at 5.8 ppm assigned to the distal isomer was almost disappeared, while the peak intensity for the proximal isomer was slightly decreased, which manifests the aza-Michael addition of AL to the distal isomer predominated over the proximal isomer probably due to the smaller steric hindrance around the alkenyl moieties.

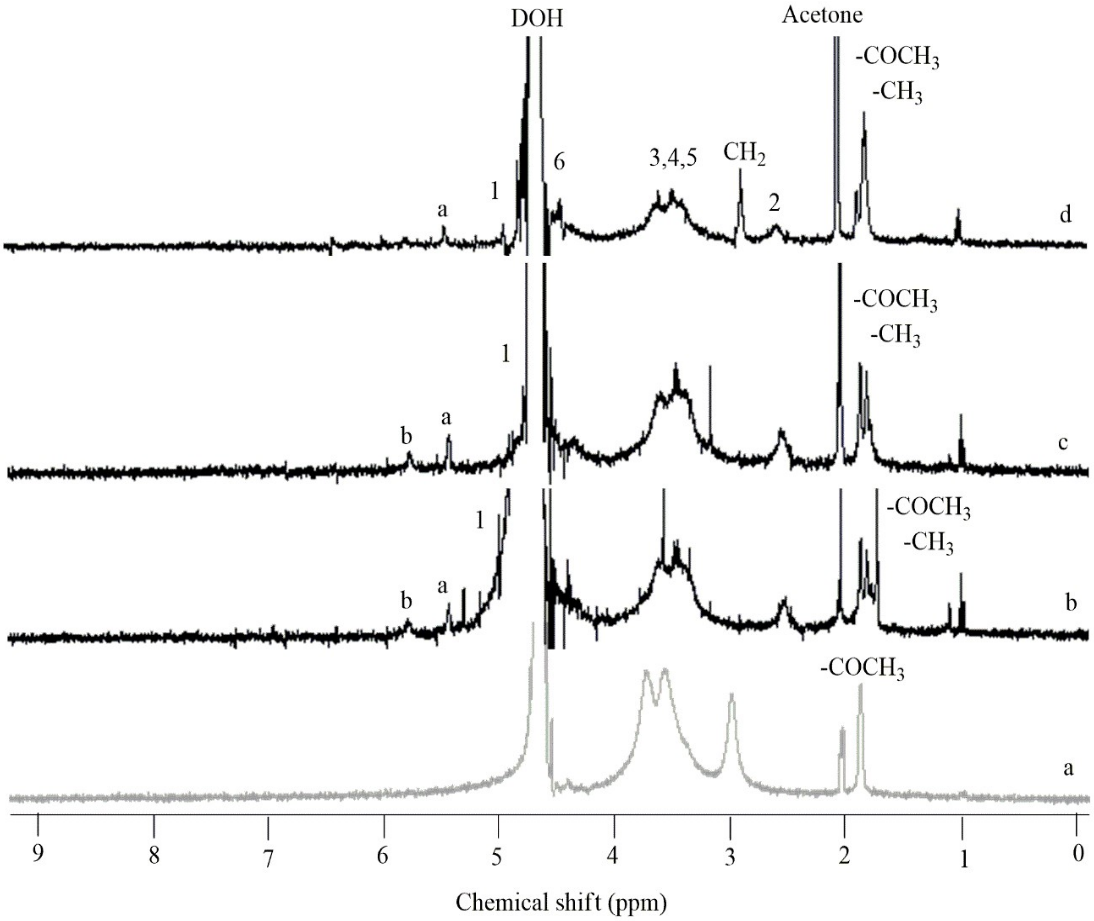

Figure S4: ^{1}H NMR spectra of (a) CS, (b) NCCS, (c) NCCS-FITC, and (d) NCCS-FITC-AL (D_2O, 400 MHz).

Fig. S5 shows the UV-visible spectra of NCCS and polyelectrolytic NCCS-FITC-AL. No significant absorption was observable in the spectrum of NCCS due to the absence of any strong chromophores in NCCS. By contrast, the spectrum of polyelectrolytic NCCS-FITC-AL shows a characteristic peak at 495 nm originating from the FITC moieties. The clear absorption without the accompanying absorption at longer wavelength resulted in the sufficient fluorescence.

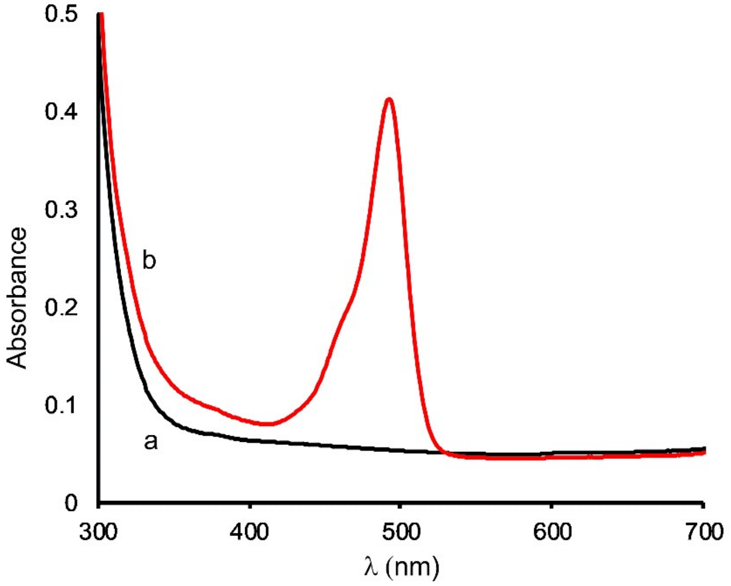

Figure S5: UV-visible spectra of (a) NCCS and (b) NCCS-FITC-AL at room temperature (H_2O, 0.35 mg•mL^{-1}).

EDX spectra analyses of Fe_3O_4, NCCS-FITC-AL, and Fe_3O_4-NCCS-FITC-AL were carried out to get information on the elemental compositions (Fig. S6$_a$). The spectrum of Fe_3O_4 shows only the signals of C, Fe, and O, and the signal of C probably arises from the carbon tape used as the support. The peaks of C, O, N, Na, and P are visible in the EDX spectrum of NCCS-FITC-AL. In the EDX spectrum of Fe_3O_4-NCCS-FITC-AL, the signals of all of these elements except Na are observed. The absence of the Na signal plausibly originates from the exchange of the counter cation of the carboxylate and phosphate moieties of NCCS-FITC-AL with the iron atoms on Fe_3O_4.

Thermal stability of Fe_3O_4-NCCS-FITC-AL nanoparticles was analysed by TGA, and compared with those of bare Fe_3O_4 and NCCS-FITC-AL as reference materials (Fig. S6$_c$). The weight loss of bare Fe_3O_4 is 6% at 400 °C and stayed constant until 800 ˚C, being assignable to the elimination of physically adsorbed and chemically dehydrated water. The thermogram of NCCS-FITC-AL shows a two-stage weight loss. Below 250 °C, the polyelectrolyte loses 7% weight for adsorbed water, and loses 55% weight from 250 to 550 °C because of the degradation of the polymer chain.[10] The residue is the carbonized product. The weight loss reaches a steady state, and the final weight loss at 800 ˚C is 58%. The TG profile of Fe_3O_4-NCCS-FITC-AL nanoparticle shows three-stage weight loss. The first 4% loss below 200 °C is due to the adsorbed water and the next 20% weight

loss below 400 °C is ascriable to the degradation of the coating material of Fe_3O_4-NCCS-FITC-AL. The weight loss from 400 to 800 ˚C was 12%, while the weight losses of Fe_3O_4 and NCCS-FITC-AL in this range are negligible. This weight loss of Fe_3O_4-NCCS-FITC-AL probably originates from the degradation of Fe_3O_4 into γ-Fe_2O_3 and FeO catalyzed by the residue of the coating material in a similar manner with carboxy-CS stabilized Fe_3O_4.[9,10]

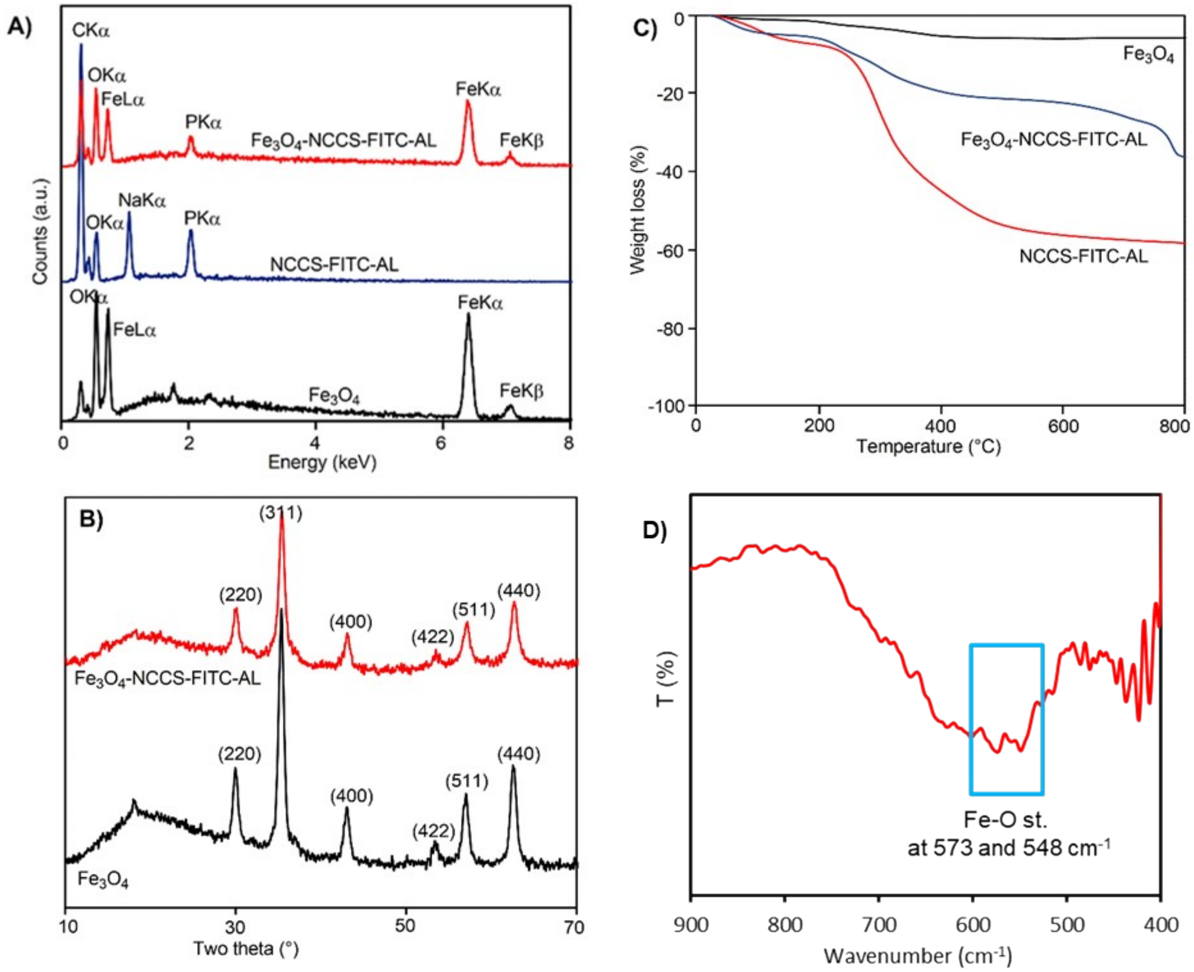

Figure S6: (A) EDS profiles (A) of bare Fe_3O_4, NCCS-FITC-AL, and Fe_3O_4-NCCS-FITC-AL nanoparticles; (B) XRD patterns of bare Fe_3O_4 and Fe_3O_4-NCCS-FITC-AL nanoparticles; (C) TGA profiles of bare Fe_3O_4, NCCS-FITC-AL, and Fe_3O_4-NCCS-FITC-AL (10 °C min^{-1}, N_2); and (D) FTIR spectrum of Fe_3O_4-NCCS-FITC-AL (900-400 cm^{-1}).

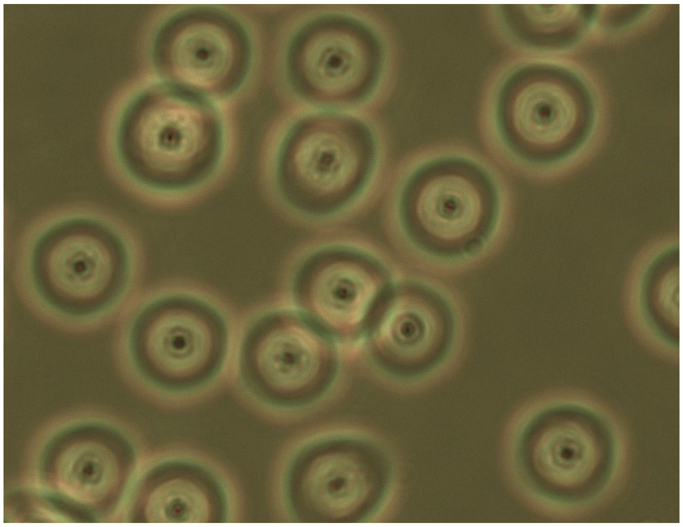

Figure S7: Light microscopic image of the negative control sample of the erythocytes dispersed in saline incubated at pH 7.4 and 37 °C (magnified under 20x level).

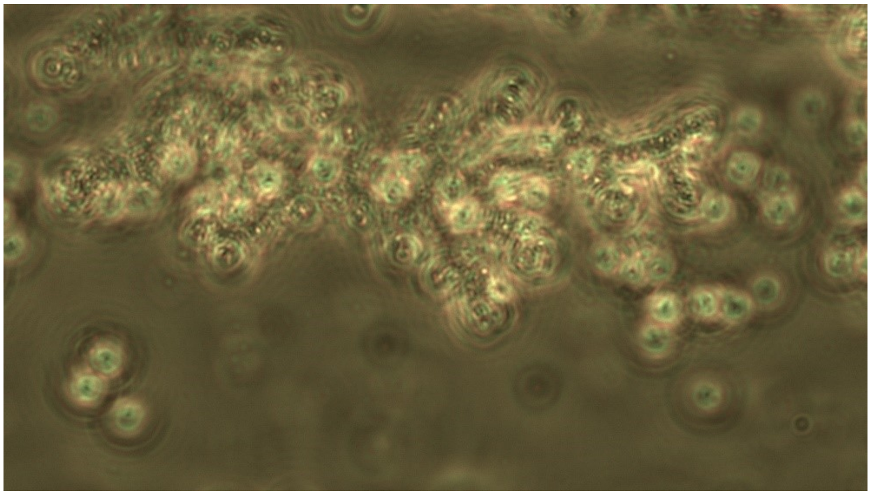

Figure S8: Light microscopic image of sheep erythrocytes agglutinated and membranoid by Fe_3O_4-NCCS-FITC-AL at pH 5.0 (100 µL mL^{-1}, 400 µg mL^{-1}, incubation time = 5 h, 37 °C).

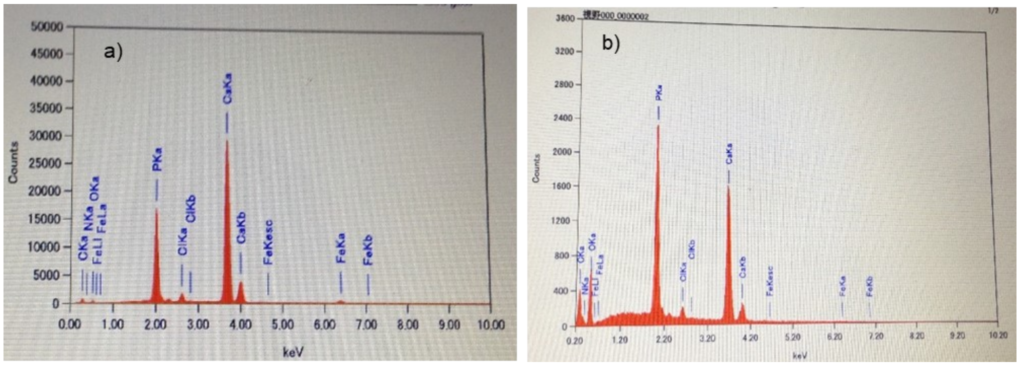

Figure S9: EDS profiles of iron content (%) in the HAp sample at pH (a) 5.0 and (b) 7.4 (Incubation time 3 h, 37 °C).

References

1. L. Alupei, A. C. Peptu, A. M. Lungan, J. Desbrieres, O. Chiscan, S. Radji, M. Popa, *Int. J. Biol. Macromol.* 2016, **92**, 561–572.
2. A. Shrifian-Esfahni, T. M. Salehi, M. Nasr-Esfahni, E. Ekramian, *Chemik* 2015, **69**, 19–32.
3. A. Dolatkhah, L. D. Wilson, *ACS Appl. Mater. Interface* 2016, **8**, 5595–5607.
4. H. M. Lee, M. H. Kim, Y. Yoon, W. H. Park, *Mar. Drugs* 2017, **15**, 105–113.
5. A. Walter, A. Garofalo, P. Bonazza, F. Meyer, H. Martinez, S. Fleutot, C. Billotey, J. Taleb, D. Felder-Flesch, S. Begin-Colin, *ChemPlusChem* 2017, **82**, 647–659.
6. A. Panahifar, M. Mahmoudi, M. R. Doschak, *ACS Appl. Mater. Interfaces* 2013, **5**, 5219–5226.
7. D. Vllasaliua, L. Casettari, R. Fowler, R. Exposito-Harris, M. Garnett, L. Illuma, S. Stolnika, *Int. J. Pharm.* 2012, **430**, 151–160.
8. H. Rezvani Alanagh, M. E. Khosroshahi, M. Tajabadi, H. Keshvari, *J. Supercond. Nov. Magn,* 2014, **27**, 2337–2345.
9. M. A. Rahman, B. Ochiai, *Microsyst. Technol.* 2018, **24**, 669 –681.
10. M. A. Rahman, Y. Matsumura, S. Yano, B. Ochiai, *ACS Omega* 2018, **3**, 961-972.
